# Chemically Etched Prussian Blue Analog–WS_2_ Composite as a Precatalyst for Enhanced Electrocatalytic Water Oxidation in Alkaline Media

**DOI:** 10.1021/acs.inorgchem.3c02537

**Published:** 2023-08-23

**Authors:** Poulami Mukherjee, Krishnamoorthy Sathiyan, Ronen Bar-Ziv, Tomer Zidki

**Affiliations:** † Department of Chemical Sciences and the Centers for Radical Reactions and Material Research, Ariel University, Ariel 4077625, Israel; ‡ Department of Chemistry, Nuclear Research Centre, Negev, Beer-Sheva 84190, Israel

## Abstract

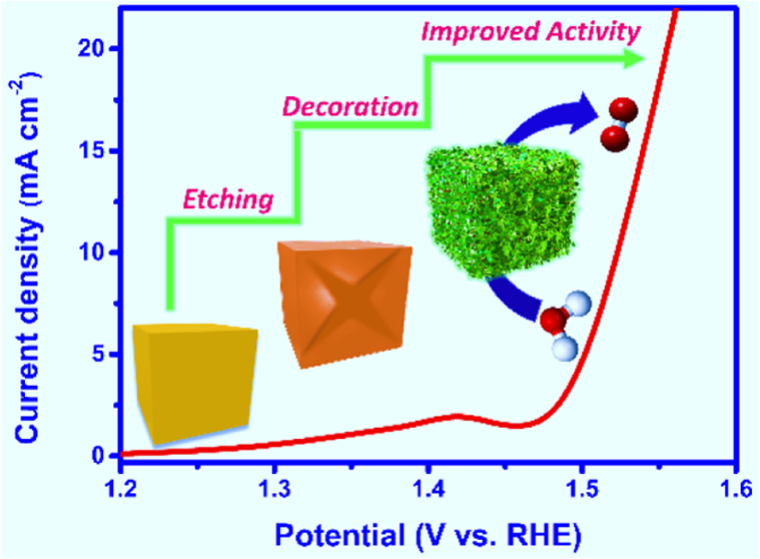

The electrochemical water-splitting reaction is a promising source of ecofriendly hydrogen fuel. However, the oxygen evolution reaction (OER) at the anode impedes the overall process due to its four-electron oxidation steps. To address this issue, we developed a highly efficient and cost-effective electrocatalyst by transforming Co–Fe Prussian blue analog nanocubes into hollow nanocages using dimethylformamide as a mild etchant and then anchoring tungsten disulfide (WS_2_) nanoflowers onto the cages to boost OER efficiency. The resulting hybrid catalyst-derived oxide demonstrated a low overpotential of 290 mV at a current density of 10 mA cm^–2^ with a Tafel slope of 75 mV dec^–1^ in 1.0 M KOH and a high faradaic efficiency of 89.4%. These results were achieved through the abundant electrocatalytically active sites, enhanced surface permeability, and high electronic conductivity provided by WS_2_ nanoflowers and the porous three-dimensional (3D) architecture of the nanocages. Our research work uniquely combines surface etching of Co–Fe PBA with WS_2_ growth to create a promising OER electrocatalyst. This study provides a potential solution to the challenge of the OER in electrochemical water-splitting, contributing to UN SDG 7: Affordable and clean energy.

## Abstract 2

A highly efficient and cost-effective electrocatalyst platform for OER was synthesized by transforming Co−Fe Prussian blue analog nanocubes into hollow nanocages using the DMF mild etchant and anchoring WS_2_ nanoflowers onto the cages. The catalyst-derived oxide demonstrated a low overpotential of 290 mV at 10 mA cm^−2^ with a Tafel slope of 75 mV dec^−1^ and a high faradaic efficiency of 89.4% in 1.0 M KOH.

## 1 Introduction

Electrochemical water-splitting is a promising method for producing green hydrogen, a sustainable and pollution-free fuel, satisfying the ever-increasing energy demand, and complying with *UN SDG 7: Affordable and cleanenergy*.^1^ Hydrogen gas can be produced in a water electrolyzer consisting of a hydrogen evolution reaction (HER), 2H_2_O + 2e^–^ → H_2_ + 2OH^–^, at the cathode and an oxygen evolution reaction (OER), 4OH^–^ → O_2_ + 2H_2_O + 4e^–^, at the anode.^2,3^ The OER is inherently slower than HER as it involves several proton–electron-coupled steps^4^ with considerable energy barriers that account for most energy losses in the electrochemical water-splitting process.^5,6^ The state-of-the-art OER catalysts are IrO_2_ and RuO_2_, but their high cost and scarce reserves hinder their large-scale sustainable applications.^6−9^ Therefore, it is of prime importance to design a promising nonprecious-metal-based OER electrocatalyst having reduced electrical resistance and improved catalytic activity.

Porous coordination polymer assemblies, including metal–organic frameworks and covalent-organic frameworks, are major candidates to serve as electrocatalysts.^10−15^ Recent progress in the upgraded design strategies of the cubical Prussian blue analogs (PBAs)—a branch of the metal–organic framework compounds, where transition-metal ions are connected with cyanide ligands—holds their unique advantages, such as several redox centers for advanced catalysis, including OER.^4,16−18^

The OER activities of the transition-metal (TM)-based catalysts highly depend on the composition, morphology, transition metal’s electron number, and the surface binding energy of oxygen.^19,20^ Co–Fe PBA demonstrates superior properties owing to the substantial charge transfer between Fe and Co due to the enhanced acidity of the Fe sites promoting M=O and M–O–O–M′ intermediate formation, which are essential for the OER process. Moreover, the weakened acidity of the Co sites eases the M–O bond cleavage of the stable M–O–O intermediate, resulting in rapid oxygen release.^21^ Metal oxyhydroxides are often the electro-oxidized derivatives of transition-metal-based (especially cobalt, nickel, and iron) precatalysts that make up the active sites through electrochemical activation by oxidation and surface reconstruction before the electrocatalytic reaction takes place;^22,23^ therefore, the catalyst can be attributed as a precatalyst.^24^ Very recently, Menezes et al. eagerly claimed that cobalt–iron oxyhydroxides (CoFeOOH_*x*_) are the most active catalysts for the OER.^25^ Galán-Mascarós et al. also reported several pioneering studies of the OER activity related to Co–Fe PBA.^26−29^ The coordinatively unsaturated Co and Fe centers would favor highly active Co/Fe-based (oxy)hydroxide layer formation, improving the affinity toward the reaction intermediates.^17^

However, the PBAs’ structural features significantly impair their catalytic performance. Their high crystallinity and low mechanical resistance are attributed to their poor interfacial matching with the electrode surface due to active site blockage in the crystalline PBAs, which hinders the electrode–electrolyte contact interface area.^30,31^

Moreover, the PBA can easily peel off the electrode surface after several cyclic voltammetry (CV) cycles due to its poor linkage to bare electrodes. Thus, many researchers have modified the PBA composite electrodes to improve PBA adherence.^32^ Modifying the PBA heterostructures by various methods such as etching, chemical decoration, doping, or transforming them into distinctive structures while maintaining their basic cubic structure is essential for improving their OER performance. These efforts are devoted to solving stability issues, exposing active sites, tuning electronic properties, accelerating electron/proton transfer, enhancing the accessible surface area, and optimizing the adsorption energy of the reaction intermediates.^33^ Compared to conventional TM oxides, PBAs provide greater synthetic control over their composition and structure.^34,35^ Their adaptability enables the precise tuning of material properties and the optimization of PBAs for specific electrochemical applications, thereby improving performance and efficiency.^36,37^ Notably, the well-coordinated water molecules in the PBA structure can also significantly lower the proton/electron-transfer energy barrier.^38^ Due to the higher intrinsic activity compared to many typical transition-metal oxides, electrochemically PBA-derived metal oxides often outperform TM oxides.^37^ Other significant limitations for TM oxide electrocatalytic applications are poor dispersion, stability, and complicated synthesis methods.^39^ Chemical etching is widely used to generate sophisticated surface topologies in PBAs with unique physical properties for diverse applications. The etchant and cube types dictate the position and path of the etching process.

Within the above modifications, introducing sulfide- or phosphide-based materials to produce PBA–hybrid structures may present a simple and effective strategy to address the PBA limitations.^40^ These derivatives can be oxidized during the OER process, resulting in amorphous metal hydroxide/oxide formation, leading to high performance and stability of the PBA.^16,41−43^ Furthermore, it is well known that metal hydroxides/oxides derived from sulfide- or phosphide-based precatalysts display better catalytic performance than the metal hydroxides/oxides synthesized directly.^22,43−45^ Growing low-dimensional structures, such as transition-metal dichalcogenide (TMD) on PBA, may alter the overall electronic structure and increase the specific surface area of the subsequent composite,^46^ boosting the charge-transfer rate and optimizing the adsorbate binding energy, thus regulating the intrinsic electrocatalytic activity of the catalyst.

Inspired by the hybrid structures and cube modification advantages, we propose a facile strategy to develop uniform Co–Fe PBA nanocages using PBA nanocubes as sacrificial templates. We introduce *N*,*N*-dimethylformamide (DMF) as an effective new etchant to create vacancy—a common form of defects^47^ in the nanocubes. This work is based on our prior report in which we employed DMF as a reducing agent (to reduce Mo^VI^ to Mo^IV^) and got some indications of its etching capabilities.^48^ This work proves that ammonia-containing species from the slow decomposition of DMF dissolve the Co/Fe ions. Different reactions and diffusion rates between the edges and face-centers of the nanocubes with the ammonia-containing species induce structural transformation in the Co–Fe PBA nanocubes, resulting in the formation of hollow cubic structures, PBA(cage).

Furthermore, we decorated the PBA(cage) structures with ultrathin TMD nanosheets such as WS_2_ to provide additional active sites. With all of these alterations, we suggest a novel approach toward the directional construction of OER active sites using DMF-etched PBA as the sacrificial template to form the metal–S precatalyst, which later undergoes electrochemical activation during the OER process. Our work proposes a unique design strategy for creating a promising OER catalyst by integrating two inert OER materials, namely, PBA and WS_2_. The composite tailored structure promotes synergism that enhances electrochemical transport due to a well-defined nanostructure framework and high conductivity.

## 2 Experimental Section

### 2.1 Chemicals and Reagents

Ammonium tetrathiotungstate [(NH_4_)_2_WS_4_] was obtained from Loba Chemie. Sodium citrate dihydrate (C_6_H_5_Na_3_O_7_·2H_2_O), potassium ferrocyanide K_3_[Fe(CN)_6_], cobalt nitrate hexahydrate (Co(NO_3_)_2_·6H_2_O), and Nafion solution (5% w/w in water/1-propanol mixture) were purchased from Alfa Aesar. The chemicals were used as received without further purification. Millipore water (deionized water, DI) with resistivity >15 MΩ·cm was used throughout the experiments.

### 2.2 Material Synthesis

#### 2.2.1 Synthesis of Co–Fe PBA Nanocubes

The Co–Fe PBA cubes were synthesized using a slight modification by the coprecipitation method adopted from the previously reported literature.^49^ Typically, solution A was prepared by mixing 0.6 mmol of Co(NO_3_)_2_ and 1.34 mmol of sodium citrate in 20 mL of DI water. Solution B, containing 0.4 mmol of K_3_[Fe(CN)_6_], was dissolved in 20 mL of DI water. Solution B was then added to solution A under stirring for 15 min to disperse the particles uniformly. The resulting mixed solution was aged for 24 h at room temperature. The final product was collected by centrifugation, washed three times with DI water and absolute ethanol, and dried at 70 °C overnight.

#### 2.2.2 Synthesis of Co–Fe PBA Nanocages

The following procedure is used to modify the as-prepared PBA cubes: 60 mg of the PBA sample was mixed with 25 mL of DMF, followed by 15 min of sonication to obtain a homogeneous solution. The solution was then transferred into a 50 mL Teflon-lined stainless steel autoclave and set for a hydrothermal reaction of varying durations at 180 °C. After cooling to room temperature, the products were collected by centrifugation, and the post-treatment was the same as above. The green catalyst obtained was labeled as PBA(cage).

#### 2.2.3 Growing WS_2_ Nanoflowers on PBA(cage) Nanocages

The main catalyst, PBA(cage)-WS_2_, was prepared by adding 20 mg of (NH_4_)_2_WS_4_ and 60 mg of PBA(cage) in 25 mL of DMF solution. It was then sonicated for 15 min and set for a hydrothermal reaction for 10 h at 180 °C. The sample was labeled as PBA(cage)-WS_2_.

The PBA(cage)-S synthesis process was similar to the above method except for replacing (NH_4_)_2_WS_4_ with thioacetamide.

#### 2.2.4 Growing WS_2_ Nanoflowers on PBA Nanocubes

As a control sample, WS_2_ nanoflowers were also grown directly on previously synthesized PBA cubes: 20 mg of (NH_4_)_2_WS_4_ was added to 60 mg of the prepared PBA sample, mixed with 25 mL of DMF, followed by 15 min of sonication. The mixture was then transferred into a 50 mL Teflon-lined stainless steel autoclave and set for a 10 h hydrothermal reaction at 180 °C. The sample was denoted as PBA-WS_2_.

#### 2.2.5 Growing Bare WS_2_ Nanoflowers

Bare WS_2_ nanoflowers were synthesized by mixing 25 mg of (NH_4_)_2_WS_4_ with 25 mL of DMF and setting it for the hydrothermal reaction under the same reaction conditions for 24 h. DMF was used as a solvent to reduce W^VI^ to W^IV^.^48^ The post-treatment was performed similarly to obtain the final product labeled as WS_2_.

#### 2.2.6 Characterizations and Electrochemical Measurements

The material characterizations and electrochemical techniques of assessment are described in the Supporting Information.

## 3 Results and Discussion

### 3.1 Structural Characterization

**Scheme 1:**
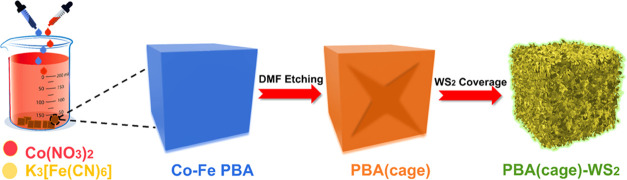
Morphological Evolution of PBA(cage)-WS_2_ Hybrids

We first synthesized the Co–Fe PBA nanocube and then etched it using DMF to produce a cage structure that was subsequently adorned with ultrathin WS_2_ nanosheets to obtain a hollowed structure composite. The morphological evolution of PBA nanocubes to PBA(cage)-WS_2_ hybrids is illustrated in Scheme 1.

**Figure 1:**
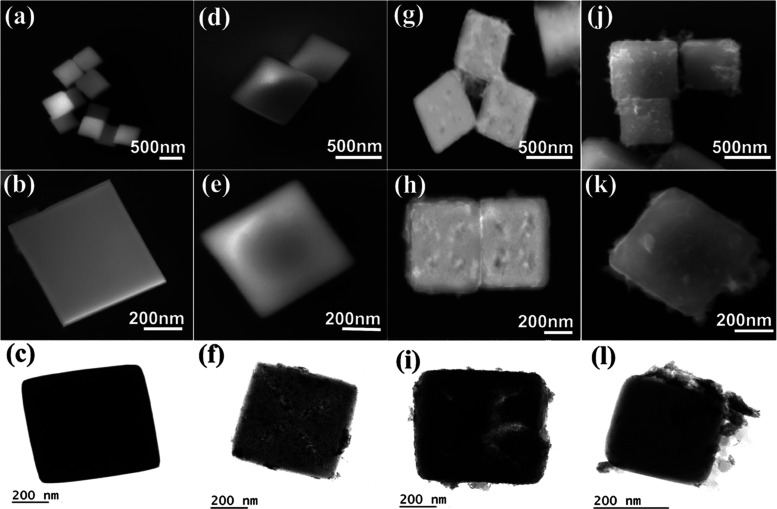
SEM images of (a, b) PBA, (d, e) PBA(cage) after 24 h etching, (g, h) PBA(cage)-WS_2_, and (j, k) PBA-WS_2_. The corresponding TEM images are in the bottom row (c, f, i, and l).

The detailed morphologies of the catalysts were analyzed by high-resolution scanning electron microscopy (SEM) and transmission electron microscopy (TEM). The SEM and TEM images of Co–Fe PBA present highly uniform nanocubes with a smooth surface and sharp corners and edges with an average size of ca. 400 nm (Figure 1a–c). A solid cube structure formation was confirmed by the darker TEM image, as shown in Figure 1c.

Upon DMF etching, the nanocubes were modified to hollow nanocages, namely, PBA(cage), along with a color change of the solid particles from purple to green, indicating structural transformation. The UV–vis spectral evolution depicted in Figure S1a indicates a progressive structural transformation process, exhibiting a gradual change over a period of at least 24 h. Figure 1d,e shows that the PBA(cage) still preserves its cubical structure after 24 h of etching. We chose a 24 h etching process as an optimum to eliminate cage breakage and to achieve a significant catalytic activity improvement, as seen from the PBA(cage) LSV curve that is discussed later. As explained in the Supporting Information (SI), Figure S1b,c proves that the spectral evolution demonstrated in Figure S1a resulted from the structural evolution of the PBA(cage) upon the etching process. The PBA(cage) SEM images confirmed the retention of the cubical nature of the nanocubes but not the formation of any hollow structure, implying that the etching process occurred mainly at the nanocube center (Figure 1d,e). Moreover, the average cube size was not changed significantly, indicating that the etching occurred only at the body center of the nanocubes. The diagonal hollow structure formation was seen from the TEM image (Figure 1f). This unique etching pattern benefited the PBA(cage) with improved energy storage performances by selectively exposing active sites and enhancing the accessible surface area.^48^

The loose inner regions generated in the nanocubes during their growth resulted in numerous packing defects.^50^ The DMF chemical etching progressed anisotropically along these loose regions, determining the final architecture. The nanocage was thus formed by removing the etchant-vulnerable Co and Fe ions from the cube center.^51^ Han et al. suggested that etching with ammonia reduces the central cross section of the cube until it disappears, forming a complete hollow nanocage within 24 h.^52^ Further etching leads to cube breakage and, ultimately, activity loss.^53^ Here, we successfully controlled the etching rate by using DMF as a milder etchant, which prevented cube breakage.

**Figure 2:**
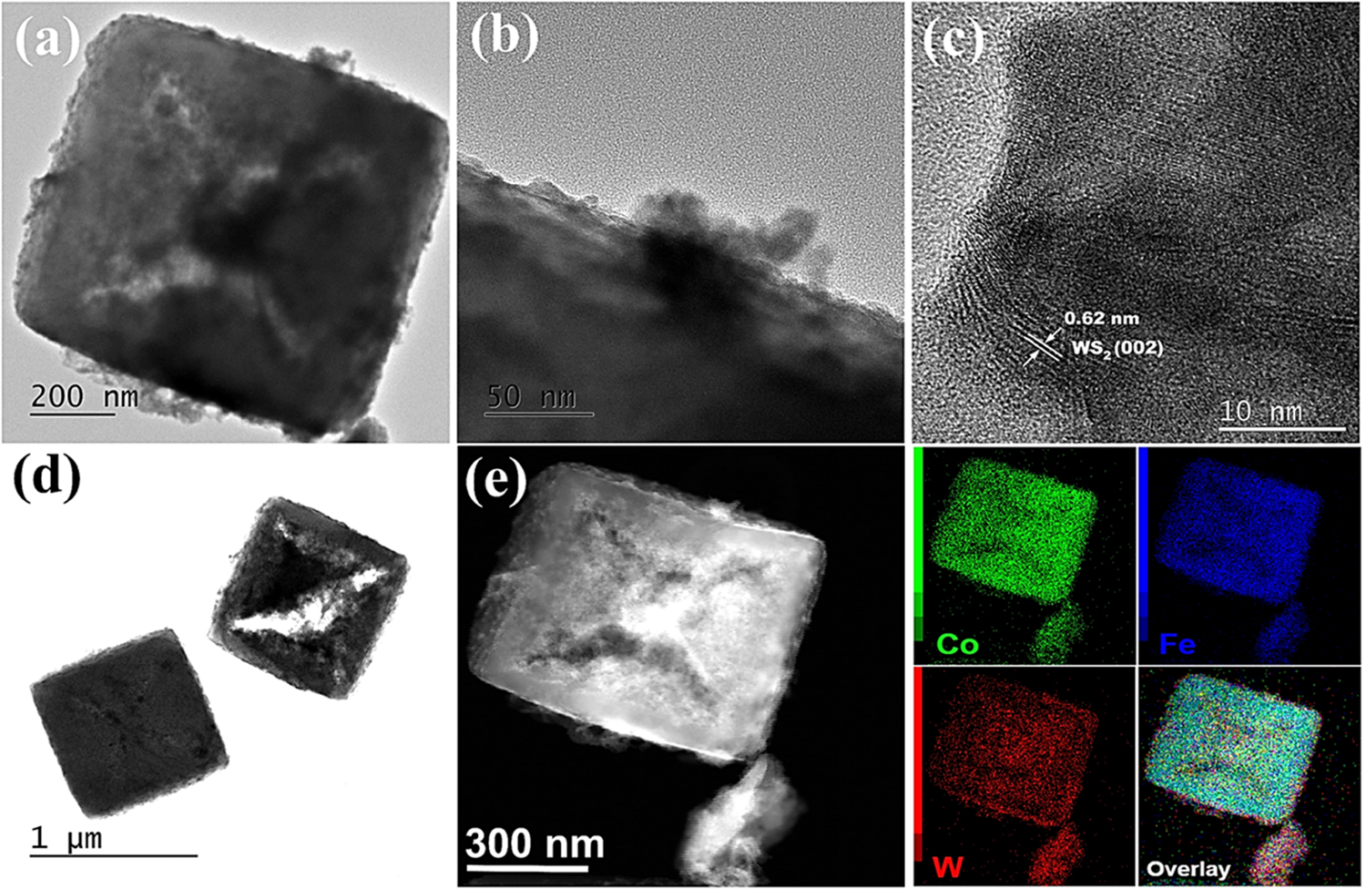
(a, b, d) TEM and (c) high-resolution (HR)-TEM images of PBA(cage)-WS_2_; (e) high-angle annular dark-field scanning transmission electron microscopy (HAADF-STEM) image and its energy-dispersive X-ray spectroscopy (EDX) elemental mapping images of PBA(cage)-WS_2_.

Next, the PBA(cage) nanocage surface was decorated with a thin WS_2_ nanosheet layer to form a hybrid structure by hydrothermal treatment with (NH_4_)_2_WS_4_ while preserving its cubic shape (Figure 1g–i). The TEM measurement clearly differentiated the interior structure of the PBA(cage)-WS_2_ from other nonetched catalysts; compare Figure 1i with l. Note that TEM is sensitive only to thin samples of a few nanometers; hence, the void structure of PBA(cage)-WS_2_ demonstrated in Figure 2 due to low-density contrast, as opposed to the opaque cube in Figure 1c, proved the efficiency of the hollowing process as the cube-face length is 400 nm.

For comparison, the PBA-WS_2_ nanocubes were synthesized under the same hydrothermal process by replacing the PBA(cage) with PBA. The SEM images of PBA-WS_2_ (Figure 1j–l) show the coverage of WS_2_ over the cubes. The WS_2_ nanosheet growth around the PBA cube was also evident from the TEM image (Figure 1l; see also the uniform coverage of WS_2_ nanosheets in Figure S4a). As a control sample, bare WS_2_ nanoflowers were synthesized hydrothermally. The SEM images (Figure S2a,b) show the bare WS_2_ with an aggregated flowerlike architecture obtained hydrothermally from the (NH_4_)_2_WS_4_ precursor. The TEM image indicated the fine-layered nanosheet formation of these nanostructures, as shown in Figure S2c.

**Figure 3:**
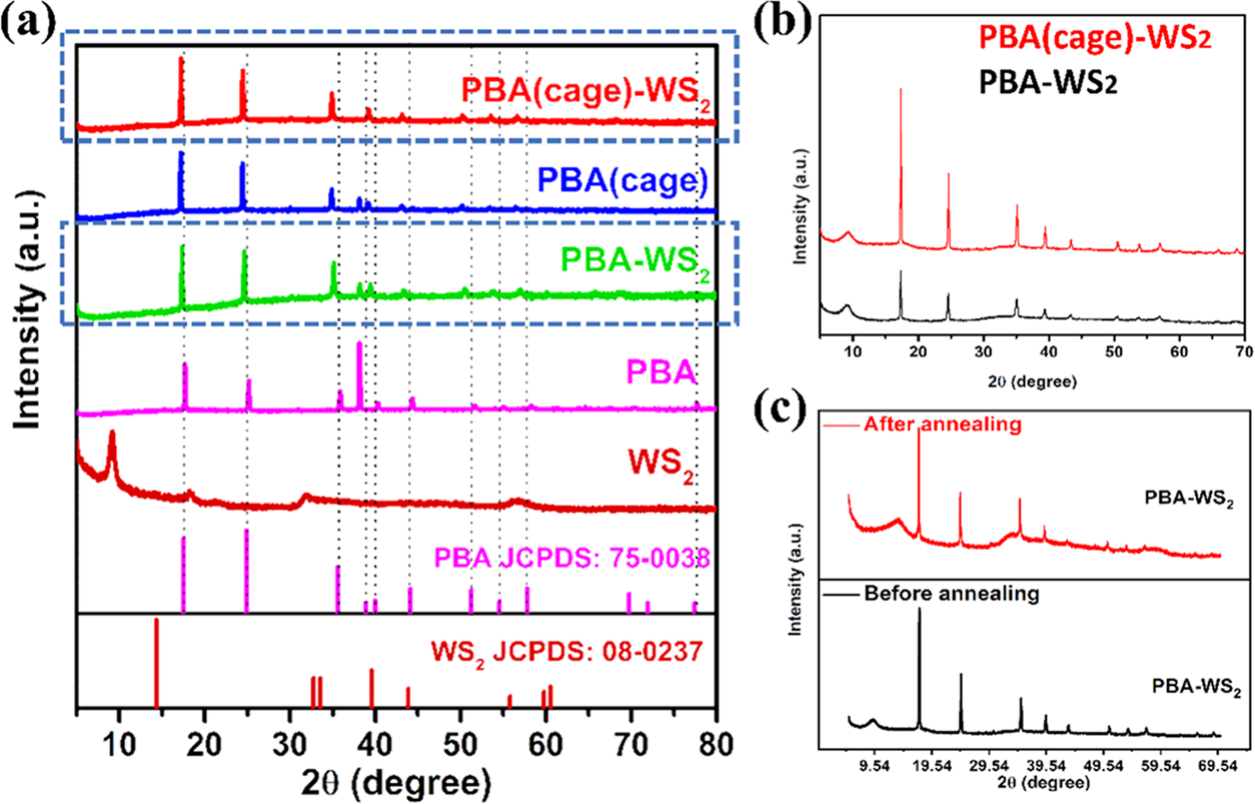
(a) XRD patterns of WS_2_, PBA, PBA-WS_2_, PBA(cage), and PBA(cage)-WS_2_; (b) slow-scan plots of PBA(cage)-WS_2_ and PBA-WS_2_; and (c) slow-scan plot of PBA-WS_2_ before and after annealing (at 350 °C for 4 h).

The crystallographic phases of the as-prepared catalysts were characterized by X-ray diffraction (XRD) spectroscopy, as shown in Figure 3a. The sharp XRD peak intensities of Co–Fe PBA at 17.6, 24.9, 35.6, 38.9, 39.9, 44.1, 51.2, 54.6, and 57.8° (attributed to (200), (220), (400), (331), (420), (422), (440), (600), and (620) crystal planes, JCDPS no. 75-0038)^54^ indicated the high crystallinity of the nanocubes with a face-centered-cubic (fcc) unit cell.^55^ No phase change was observed upon etching the PBA; however, the PBA(cage) and PBA(cage)-WS_2_ peaks were slightly downshifted relative to PBA due to the cube-to-cage structural alteration. These observations can be ascribed to defect formation during the etching process.^56^ Interestingly, in the nonetched PBA bound to WS_2_, PBA-WS_2_, XRD patterns were also downshifted compared to PBA. The source of the XRD pattern modifications could be the WS_2_ growth over PBA carried out via the short-time hydrothermal reaction in DMF, akin to the etching procedure. The slow-scan XRD patterns, shown in Figure 3b, revealed the existence of WS_2_ nanoflowers in PBA-WS_2_ and PBA(cage)-WS_2_. XRD measurement further supported the effective fabrication of the WS_2_ nanoflowers. The peaks were attributed to the hexagonal phase of WS_2_ and corresponded to the standard WS_2_ pattern, JCPDS no. 08-0237.^57^ The increased interlayer spacing, attributable to the intercalation of the DMF solvent between the WS_2_ layers,^58^ caused the (002) diffraction peak of WS_2_, typically at a 2θ of 14°, to move to a lower Bragg angle of 9.1°.^59^ Also, the crystallinity decreased due to the formation of the layered structure, ascribed to the lower and broader peaks. The WS_2_ (002) peak in PBA-WS_2_ shifted back to 14° after annealing, demonstrating that the DMF solvent intercalated between its interlayers rather than between Co and Fe (Figure 3c).

The growth duration of WS_2_ on the PBA(cage) was varied between 5 and 24 h to track the catalytic activity dependence on the WS_2_ thickness. Figure S3a,c displays SEM images of PBA(cage)–WS_2_ at various reaction durations, indicating that the formation of the nanosheets was consistent with the reaction time. Five hours were insufficient for the growth of the WS_2_ on the PBA(cage). However, after 24 h, WS_2_ nanosheet overgrowth on the surface of the cages was observed, covering their active sites and resulting in a loss of catalytic performance (see Section 3.2). It was discovered that a 10 h growing period was ideal for the nanoflower decoration of nanocages; this was also electrochemically confirmed (see Section 3.2).

The HR-TEM image (Figure 2c) shows the lattice fringes of the WS_2_ nanoflowers grown on PBA(cage). With a lattice *d*-spacing of 0.628 nm, these fringes matched the interplanar distance of the (002) plane.^59^ As confirmed by XRD, the larger *d*-spacing than standard WS_2_ (0.615 nm) resulting from DMF or its decomposition species (dimethyl amine) intercalation in WS_2_ interlayers is advantageous for energy storage applications.^59^ The dark-field scanning TEM (STEM) image and associated energy-dispersive X-ray spectroscopy (EDX) elemental mapping of PBA(cage)-WS_2_ showed that Co, Fe, and W are evenly distributed across the nanocages, as shown in Figure 2e (and Figure S4b for PBA-WS_2_). The EDX analysis of the PBA cubes revealed a Co/Fe molar ratio of 3:2, consistent with the expected value of the Co_3_[Fe(CN)_6_]_2_ template. In contrast, the cage composition had a slightly reduced Co/Fe ratio compared to the precursor’s PBA cubes (Table S1), indicating the cobalt preference to migrate out of the cube and be soluble in DMF, establishing a cagelike framework with Co/Fe of ∼1.33. Table S2 shows the inductively coupled plasma optical emission spectroscopy (ICP-OES) analysis results. Fourier transform infrared (FTIR) spectra confirmed the coordination bonds within the PBA framework (M—N≡C—M′) and the ammonia-containing species formation in the etched PBA nanocages (Figure S5). The amount of WS_2_ in PBA(cage)-WS_2_ was determined using ICP-OES to be ∼12.5 wt % of the entire amount of PBA.

**Figure 4:**
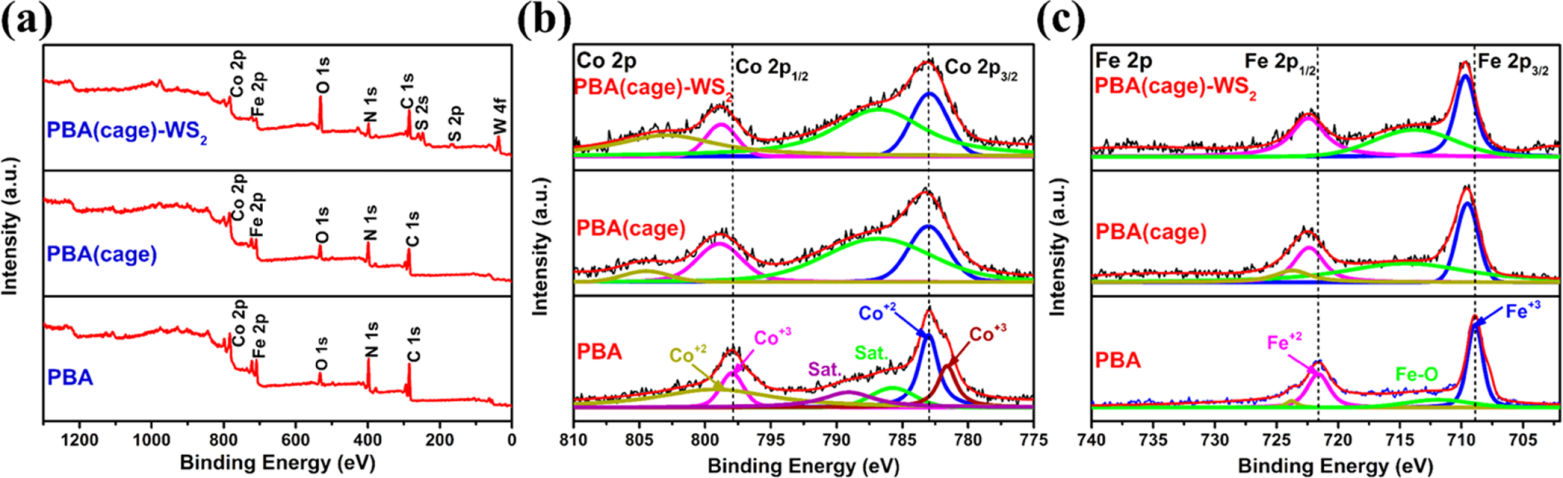
(a) XPS survey spectra of PBA, PBA(cage), and PBA(cage)-WS_2_; high-resolution XPS spectra of (b) Co 2p and (c) Fe 2p for PBA, PBA(cage), and PBA(cage)-WS_2_.

The surface chemical compositions and elemental valence states of PBA(cage)-WS_2_, PBA(cage), PBA, and WS_2_ were analyzed by X-ray photoelectron spectroscopy (XPS). The XPS survey spectra of PBA, PBA(cage), PBA(cage)-WS_2_, and WS_2_ (Figures 4a and S7a) confirmed the presence of Co, Fe, W, S, and some O from superficial oxidation due to air contact.^60^ The high-resolution Co 2p and Fe 2p XPS spectra of PBA, PBA(cage), and PBA(cage)-WS_2_ are shown in Figure 4b,c. A detailed study of the binding energies (BEs) is given in the Supporting Information file. The BE peaks of Fe 2p_1/2_ and 2p_3/2_ in PBA(cage) and PBA(cage)-WS_2_ were upshifted compared to that of PBA (Figure 4c), indicating a higher electron affinity that eases water oxidation.^60^ Combining the FTIR and XPS results, Fe^2+^ was oxidized by Co^3+^. Since the FTIR results show that some Fe^2+^ existed after etching, and because the surface-sensitive XPS showed mostly Fe^3+^ in the etched samples, we can conclude that the oxidation of Fe^2+^ to Fe^3+^ occurred mainly at the cages’ surface. Assuming that the DMF (or its product) acted like ammonia, it preferentially etched the interior Fe^3+^.^61,62^

Similarly, the W peaks upshifted in PBA(cage)-WS_2_ compared to bare WS_2_ (Figures S6a and S7b). Hence, the thin WS_2_ layer on the PBA(cage) promoted OER catalysis. High-resolution XPS spectra of S in PBA(cage)-WS_2_ and bare WS_2_ are shown in Figures S6b and S7c. Consequently, the apparent signal shifts of all of the elements in PBA(cage) and PBA(cage)-WS_2_ upon etching and WS_2_ growth validated the interactions and synergism between the catalyst’s components.

### 3.2 Electrochemical OER Performance of PBA(cage)-WS_2_

**Figure 5:**
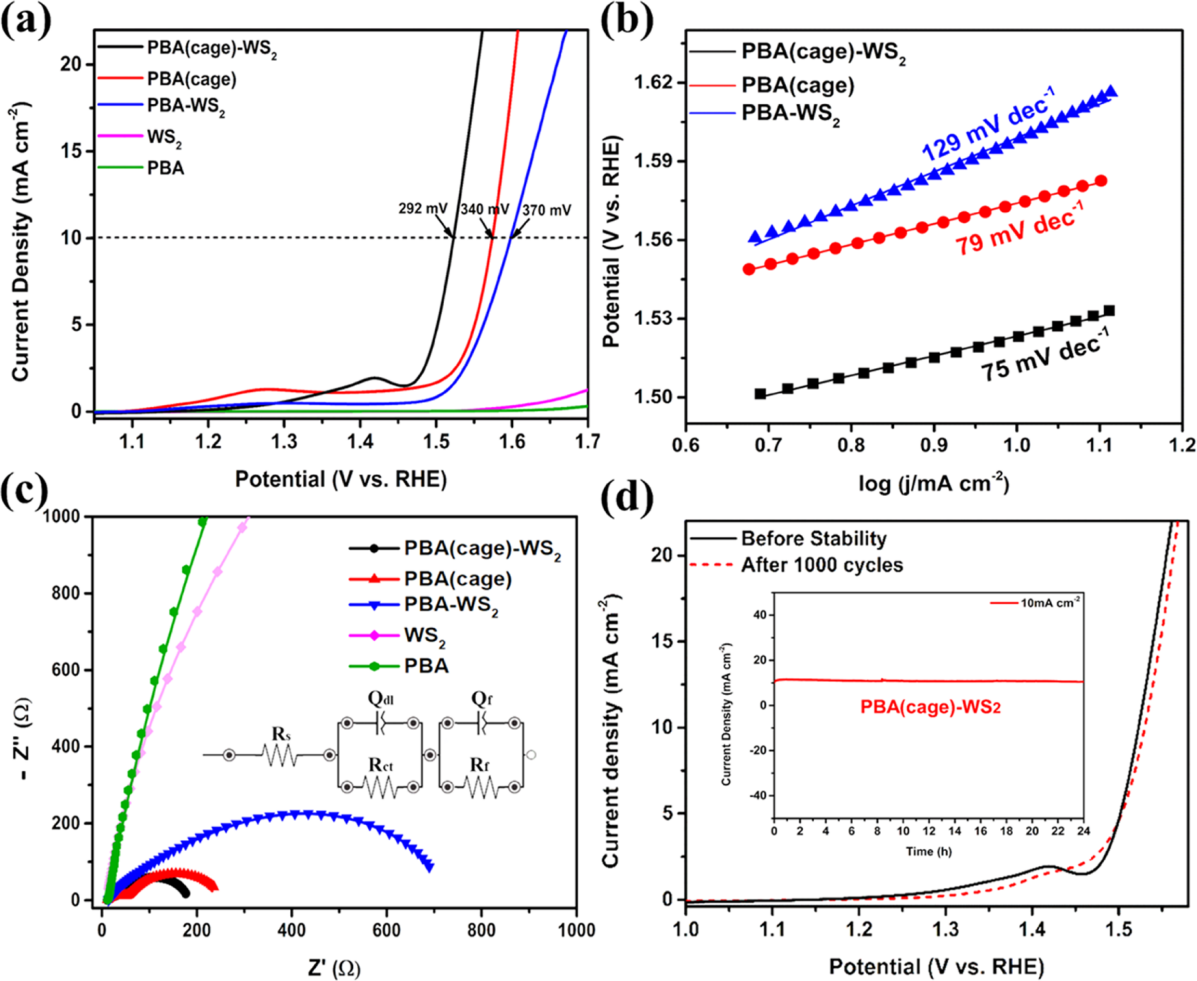
Electrochemical study of the different catalysts: (a) LSV curves in 1.0 M KOH at 10 mV s^–1^; (b) corresponding Tafel plots; (c) electrochemical impedance spectra (EIS)–Nyquist plots measured at 1.52 V vs RHE in 1.0 M KOH with the equivalent circuit; and (d) OER stability test–LSV curves of the PBA(cage)-WS_2_ before (solid black) and after (dashed red) 1000 CV cycles; the inset shows the chronoamperometric time-dependent current density curve during electrolysis at ∼1.52 V for 24 h on PBA(cage)-WS_2_.

The electrochemical performance of the PBA(cage)-WS_2_ hybrid structure was evaluated in a typical three-electrode system in a 1.0 M KOH solution (Figure 5). For comparison, the electrocatalytic activity was also tested for PBA(cage), PBA-WS_2_, PBA, and WS_2_ under the same conditions. In addition, the PBA was preoxidized in a procedure adopted from the literature to verify whether the oxidized PBA is the source of the OER activity; see the SI for further details and Figure S8. The PBA(cage)-WS_2_ achieved the current density benchmark of 10 mA cm^–2^ at an overpotential of 290 ± 3 mV, much lower than those of bare PBA(cage) (∼340 mV) and PBA-WS_2_ (∼370 mV); see Figure 5a. The potent PBA(cage)-WS_2_ showed good reproducibility in its OER performance (Figure S9 and Table S3). Note that the LSV curves are taken after preconditioning of all of the catalysts; i.e., several CV scans were run until stable CV curves were obtained. During this time, the actual active species evolution associated with electrochemical oxidation occurs.^22^ This in situ electrochemical tuning is pH-dependent and occurs very fast in a strong alkaline medium (1.0 M KOH). The first four CV cycles of PBA-WS_2_, PBA(cage), and PBA(cage)-WS_2_ are shown in Figure S10a–c. Both bare WS_2_ and PBA separately exhibited negligible OER activity. Even at high overvoltage (1.7 vs RHE), they could not reach 10 mA cm^–2^, highlighting the synergistic effect in the hybrid nanostructured catalyst. The electrochemical activity significantly increased when WS_2_ wrapped the PBA nanocubes due to the creation of active interface sites^63,64^ and the heteroatomic synergistic effect (Figure 5a). This interface effect decreased at high WS_2_-shell coverage around the cube, as shown by the LSV curves of PBA(cage)-WS_2_ at different growth durations (Figure S11a). We found that the optimal WS_2_ coverage on PBA(cage) was obtained after 10 h of growth (with a PBA/(NH)_4_WS_2_ precursor feed ratio of 3:1) (Figure S11b). The overgrowth of WS_2_ on the PBA cubes may have resulted in insulating or poorly conducting regions, which impeded electron transfer and reduced the overall activity of water oxidation. Also, if the WS_2_ layer is too thick or densely packed, then only a few water molecules could reach the active surface, leading to potential mass transportation restrictions. Moreover, this result suggests that the active site is the contact area between PBA and WS_2_. Also, the role of including W as an additional metal, enhancing the charge-transfer rate, and modulating the electronic structure to help tune the catalytic performance is shown in Figure S11c.

Moreover, the optimal hybrid PBA(cage)-WS_2_ catalyst had the lowest Tafel slope (Figure 5b), indicating faster OER kinetics; this was corroborated by its smallest semicircle diameter in the electrochemical impedance spectra (EIS; Figure 5c, where the fitting results are given in Table S4), indicating faster electron transfer on its surface during the OER. Thus, the overall image drawn from the above-mentioned activity metrics is that PBA nanocages generated by a chemical etching technique, especially when wrapped in WS_2_, are much better catalysts than both Co–Fe PBA cubes and WS_2_.

To further explore the factors controlling the OER performance of our system, using CV, we estimated the electrochemically active surface area (ECSA) directly proportional to double-layer capacitance, *C*_dl_ (Figure S12a–e).^65^ By plotting half of the difference between anodic and cathodic current densities (Δ*J* = *J*_a_ – *J*_c_) vs different scan rates, a straight line was obtained with a slope equivalent to the *C*_dl_, as shown in Figure S12f. The *C*_dl_ values for PBA(cage)-WS_2_, PBA(cage), PBA-WS_2_, PBA, and free-standing WS_2_ were 1.44, 1.28, 0.4, 0.02, and 0.05 mF cm^–2^, respectively. Table S5 summarizes the *C*_dl_ and ECSA values of all of the electrocatalysts. The most pronounced effect was the DMF etching, which generated cavities in the PBA(cage) with additional exposed active sites, increasing the ECSA by a factor of more than 60. Shelling the PBA nanocages with WS_2_ also increased the electrochemical surface area to some extent, implying that the highest electrocatalytic OER activity of the cages may be attributed to their largest ECSA, which benefited from the high electrocatalyst–electrolyte contact interface area provided by their open and hollow nanostructures.

The effect of the electrode surface area on the overall catalytic activity can be eliminated by plotting the LSV curves by normalizing the catalytic currents using ECSA (rather than the geometric area) (Figure S13). Notably, upon ECSA normalization, the PBA(cage) showed inferior activity compared to that of the unetched PBA-WS_2_. The latter even merged with PBA(cage)-WS_2_, emphasizing the synergistic effect of the hybrid structure and contributing to the higher intrinsic activity of each accessible site. The amount of oxygen produced during the electrolysis of the OER at 10 mA cm^–2^ was measured using the PBA(cage)-WS_2_ catalyst to confirm that the observed oxidation currents were indeed associated with water oxidation. The faradaic efficiency (FE) was calculated by comparing the actual amount of oxygen produced by the potentiostatic anodic reaction to the theoretical amount assuming 100% FE.^66^ The obtained FE for PBA(cage)-WS_2_ was approximately 89.4%, as shown in Figure S14. This result provides strong evidence that the observed oxidation currents indicate water oxidation.

Stable activity and long-term durability are critical characteristics for the commercial application of the catalyst.^52^ Even after 24 h, no significant change in activity could be observed for the PBA(cage)-WS_2_ catalyst at a current density of 10 mA cm^–2^ (inset of Figure 5d). Moreover, the LSV polarization curves of PBA(cage)-WS_2_ before and after 1000 CV cycles at potentials ranging from 1.0 to 1.7 V (vs RHE) showed a stable onset OER potential and current density; yet, the oxidation peak vanishes upon continuous cycling, revealing the irreversible in situ electrochemical catalyst tuning (oxidation) forming OER-active metal (oxy)hydroxides under the OER conditions (Figure 5d). The preoxidized PBA demonstrated an improved OER activity compared to normal PBA with an overpotential of 410 mV at a current density of 10 mA cm^–2^, indicating that the electrochemical oxidation produced active catalytic sites; still, the hybrid PBA(cage)-WS_2_ exhibited a much higher OER activity, signifying the synergism between the hybrid components.

**Figure 6:**
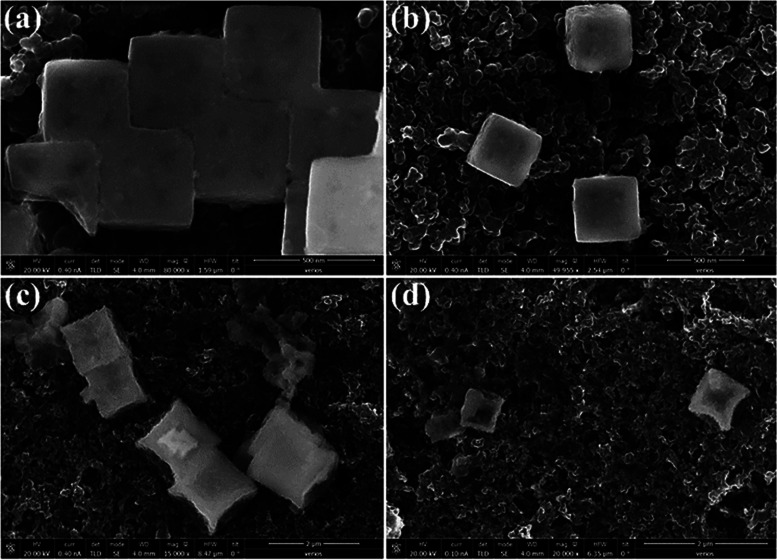
HR-SEM images of PBA(cage)-WS_2_ (a, b) before (c, d) after 1000 cycles.

The SEM images before and after the catalytic process showed that the catalyst maintained its original cubic framework (Figure 6). Still, a minor deformation of the cubes with an ultrathin nanosheet coverage indicated the structural evolution and formation of CoFeO_*x*_ (Figure 6c,d) consistent with the EDX data, demonstrating a drop in the metal content with increasing oxygen content (Table S6). The data above indicates that the catalyst was converted into oxide-based materials throughout the OER process. The XPS was also used to determine the chemical states of the catalyst following the OER. As seen in Figure S15, the XPS survey spectra showed that the O signal increased following catalysis.

Moreover, the Co 2p_3/2_ peak shifted from 781.4 to 781.8 eV, corresponding to the CoO_*x*_ formation (Table S7).^67^ The prominent peaks of O 1s spectra at 532.2 and 531.1 eV in Figure S16 are attributed to Co–OH and Fe–OH, respectively.^67^ The peaks of W and S in PBA(cage)-WS_2_ also shifted to higher BE after catalysis (Figure S17). The XPS quantification results, in Table S7, supported the EDX results. The results indicated that metal sulfides are thermodynamically unstable under oxidizing potentials and transformed into their corresponding metal oxides/(oxy)hydroxides,^68^ which serve as active species for the OER.^37^Table S8 compares the OER catalytic activities of reported pristine TM oxides and PBA-oxides with our catalyst. Our catalyst’s superior activity confirms that inducing (oxy)hydroxides through the directional construction of active sites in PBA-based precatalysts is an effective technique for the synthesis of OER catalysts.

## 4 Conclusions

In summary, we report the synthesis of hybrid PBA-WS_2_ by a sequential three-step strategy: template growth, framework etching, and subsequent decorating with WS_2_ nanoflowers. The hybrid electro-oxidized catalyst has a promising OER performance, requiring only 290 mV overpotential to attain the current density benchmark of 10 mA cm^–2^, which is superior to all of its components. This study emphasized the importance of using DMF as a mild etchant in the controlled transformation of nanocubes to hollow nanocage structures. Listed below are the two key findings from the study: (1) hollow porous architecture is required for the optimized active site exposure, speeding up electron transfer and inducing a high specific surface area that promotes electrolyte infiltration; (2) WS_2_ nanoflowers can be successfully and controllably deposited on PBAs to yield hybrid nanostructures that synergistically lead to higher intrinsic activity. This work contributes to the ongoing challenge of developing cost-effective PBA-based electrocatalysts for water-splitting electrolysis.

## Supporting Information Available

Material characterization, electrochemical procedure, tabulated data of the results, and supportive experimental results (PDF)

## Abbreviations

OER: oxygen evolution reaction
Co–Fe PBA: cobalt–iron Prussian blue analog
WS_2_: tungsten disulfide
HER: hydrogen evolution reaction
TMD: transition-metal dichalcogenide
DMF: *N*,*N*-dimethylformamide
SEM: scanning electron microscope
HR-TEM: high-resolution transmission electron microscope
XRD: X-ray diffraction spectroscopy
ICP-OES: inductively coupled plasma optical emission spectroscopy
FTIR: Fourier transform infrared
XPS: X-ray photoelectron spectroscopy
LSV: linear scanning voltammetry
ECSA: electrochemically active surface area
EIS: electrochemical impedance spectra
