# Supplementary material for: Chemically Etched Prussian Blue Analog–WS2 Composite as a Precatalyst for Enhanced Electrocatalytic Water Oxidation in Alkaline Media
Source: Inorg Chem. 2023 Aug 23;62(35):14484–93. doi: 10.1021/acs.inorgchem.3c02537 (PMC10481376; doi:10.1021/acs.inorgchem.3c02537)
Supplement: Supplementary file 1 — ic3c02537_si_001.pdf [file ic3c02537_si_001.pdf]

Supporting Information

Chemically Etched Prussian Blue Analog–WS_2 Composite as a Precatalyst for Enhanced Electrocatalytic Water Oxidation in Alkaline Media

Poulami Mukherjee,[a] Krishnamoorthy Sathiyan,[a] Ronen Bar-Ziv,[b,*] and Tomer Zidki[a,*]

a Chemical Sciences Department and the Centers for Radical Reactions and material research, Ariel University, Ariel, 4077625, Israel.

b Department of Chemistry, Nuclear Research Centre, Negev, Beer-Sheva, 84190, Israel.

*Corresponding authors

E-mail: tomerzi@ariel.ac.il

bronen@post.bgu.ac.il

Material characterization

The XRD (X-ray diffraction) patterns were recorded in the 2θ range of 5-80º (step size 0.02º and 5.0 s per step) by Panalytical X'Pert Pro X-ray powder diffractometer with Cu Kα radiation (λ = 0.154 nm). The High-resolution scanning electron microscopy (HR-SEM) images were obtained by a Tescan MAIA3 equipped with an energy dispersive spectrometer (EDS) detector. Transmission electron microscopy (TEM) images were prepared by drop-casting a sonicated sample solution in a propanol-water (1:1) mixture on a carbon film-coated 300 mesh Cu grid (Tecnai 12 microscope – FEI). The TEM samples were allowed to dry overnight at room temperature for further analysis. Transmission electron microscopy images and selected area electron diffraction (SAED) patterns were acquired using a Tecnai 12 microscope (FEI) TEM and a JEM 2100, JEOL (200 kV) High-resolution TEM (HR-TEM). The produced catalysts absorption peaks were detected by a UV-vis NIR spectrophotometer (Hitachi U-4100). Fourier transform infrared (FTIR) spectra were obtained using Bruker Alpha II infrared spectrometer in a 4000-500 cm^{-1} scan range in the TR mode. The ESCALAB 250 ultrahigh vacuum (1×10^{-9} bar) apparatus with an A1 Kα x-ray source and monochromator with an X-ray beam size of 500 μm was used for obtaining XPS for all the samples. The high-resolution spectra were recorded with a pass energy (PE) of 20 eV.

Electrochemical measurements

Each catalyst ink for the working electrode was prepared by dispersing 2.5 mg of sample in a mixture of 200 µL water, 200 µL isopropyl alcohol, and 10 µL of Nafion binder, followed by ultrasonication for 15 min. Before using the glassy carbon electrode (GCE), the surface was polished with 0.3 µm and 0.05 µm of alumina powder to smoothen the surface. The GCE was then sonicated in water for 30 s using an ultrasonic bath to remove any particles, and 30 cycles of CV were performed in 0.50 M H_2SO_4 to clean it electrochemically. Finally, 10 µL of the ink (catalyst loading 0.86 mg cm^{-2}) was drop-casted on the thoroughly cleaned GCE, with a diameter of 3 mm, using a micropipette, and dried at room temperature.

The electrochemical OER activities of all the prepared catalysts were investigated in an N_2-purged 1.0 M KOH solution (pH ~14) using the PalmSens4 electrochemical workstation. A conventional three-electrode setup of a GC working, Hg/HgO reference, and graphite rod counter electrodes was used to perform the electrochemical measurements. The potential reported in this work is referenced to the reversible hydrogen electrode (RHE) using the Nernst equation $E_{RHE} = E_{Hg/HgO} + 0.059 * pH + 0.098$. The charging currents were measured from the double-layer charging curves using cyclic voltammograms (CVs) at different scan rates of 20 to 200 mV s^{-1} at a non-faradic potential range for OER where no redox process occurs. The electrochemical surface area (ECSA) is estimated from the electrochemical double-layer capacitance (C_{dl}) at the catalyst│electrolyte interface by applying a general specific capacitance of 40 µFcm^{-2}.[1,2] Electrochemical impedance spectroscopy (EIS) measurements were carried out at an overpotential of 292 mV from 100 kHz to 0.1 Hz (15 points per decade) with an amplitude of the sinusoidal perturbation fixed at 10 mV. The polarization curves are represented without iR correction. The Faradaic efficiency (FE) experiment was performed in a gas-tight H-cell connected to a manometer on the anodic compartment. When a constant potential was applied by chronoamperometry measurement, the change in the water level of the manometer was observed with time. The data recorded are converted into moles of oxygen.

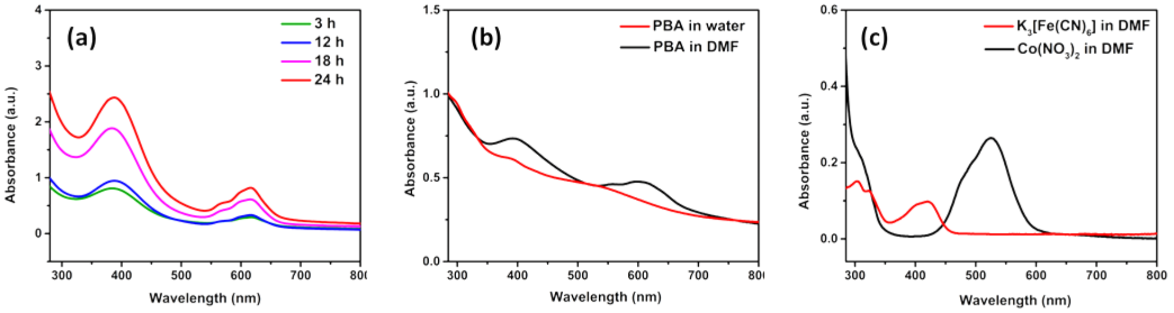

Figure S1. UV-Vis spectra of (a) PBA(cage) at various etching durations, (b) unetched PBA in water and in DMF, (c) PBA precursors in DMF. The PBA samples were treated in DMF, filtered, and resuspended in water.

Figure S1a illustrates the UV-visible absorption spectra of PBA(cage) samples subjected to varying etching durations. The solid samples were dispersed in water, and the spectra were acquired to gain insights into the structural transformation process associated with the etching procedure. **Figure S1b** shows that the source of the peaks in **Figure S1a** is the DMF adsorption on PBA, even without etching. To prove that the peak did not come from metal ions dissolution in DMF, **Figure S1c** demonstrates the PBA precursors in DMF. The peaks in **Figure S1c** differ from those of PBA in DMF, indicating that the peaks in **Figure S1a** stem solely from DMF adsorption on PBA.

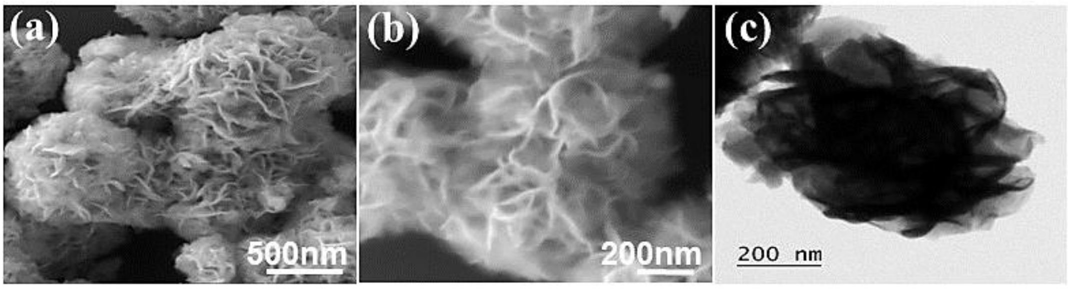

Figure S2. TEM images of WS_2 (a-b) HR-SEM, and (c) Low magnification.

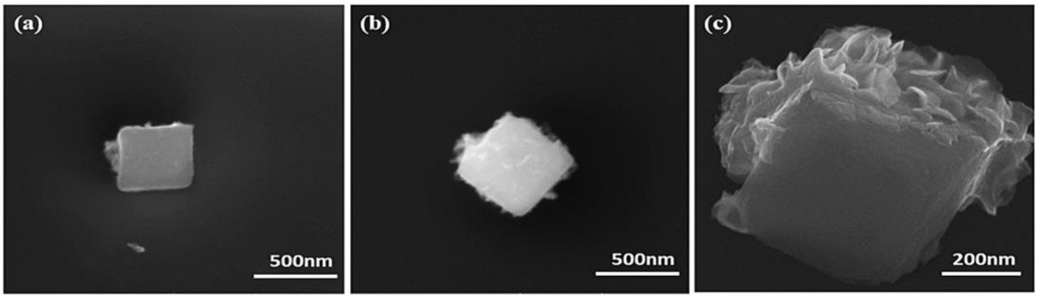

Figure S3. HR-SEM images of WS_2 growth on PBA(cage) after (a) 5 h, (b) 10 h, and (c) 24 h reaction duration.

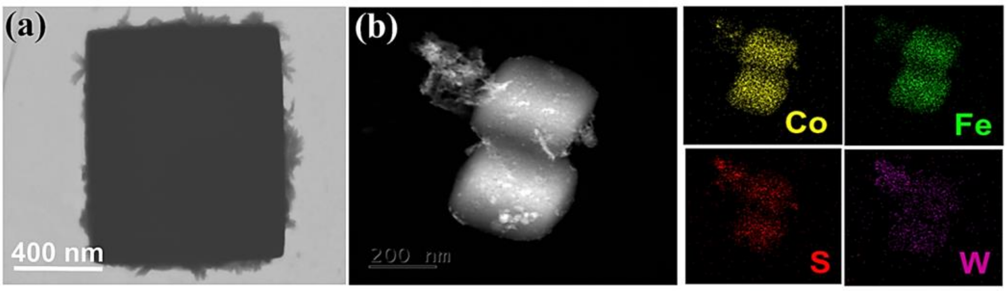

Figure S4. (a) STEM images of PBA-WS_2;(b) the corresponding elemental mapping of the same frame showing Co, Fe, S, and W.

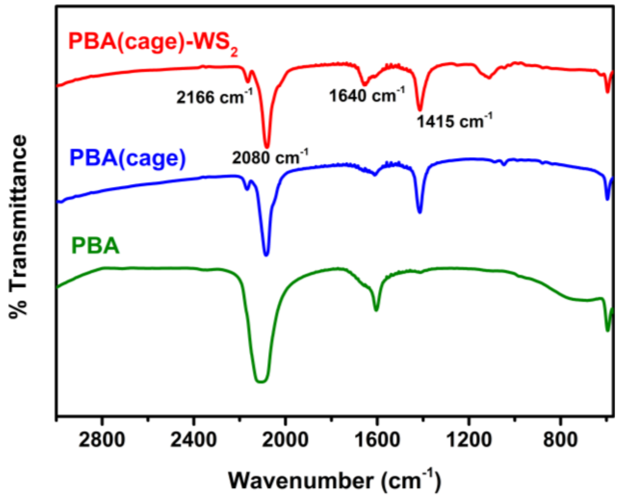

Figure S5. FTIR spectra of PBA, PBA(cage), and PBA(cage)-WS_2.

Figure S5 demonstrates the FTIR spectra of the prepared catalysts. The band centered at 2,080 cm^{-1} is assigned to the stretching frequency of the -CN group coordinated by Co^{2+} and Fe^{2+}.[3] The peak at 1,640 cm^{-1} belongs to O-H bending vibrations of absorbed water molecules.[4] In PBA(cage) and PBA(cage)-WS_2, a new band at 2,166 cm^{-1} corresponding to Co^{2+}–CN–Fe^{3+} is observed.[3] According to Nai et al., Co^{2+}–CN–Fe^{2+} and Co^{2+}–CN–Fe^{3+} co-exist in the nanocages but are inhomogeneously distributed, where the Co^{2+}–CN–Fe^{2+} sites are mainly located at the surfaces of the cages, and the Co^{2+}–CN–Fe^{3+} at the body center.[5] The 2,080 cm^{-1} peak in the PBA curve is wider than in the PBA(cage) curves. Due to the peak width, we cannot exclude the presence of Co^{2+}–CN–Fe^{3+} in the PBA. Upon etching, the peak is lower and narrower and a new Co^{2+}–CN–Fe^{3+} peak at 2,166 cm^{-1} arises. Hence, the FTIR spectra indicate smaller amount of Co^{2+}–CN–Fe^{2+} in the etched samples. The Fe^{2+} may be preferably leached of the cube or oxidized via Co^{2+}–CN–Fe^{2+} $\rightarrow$ Co^{2+}–CN–Fe^{3+}. Together with the XPS results, see below, we conclude that this oxidation takes place preferably in the cube centre. The peak at 1,415 cm^{-1} is assigned to adsorbed to N-H species, supporting the anticipation that DMF molecules decompose to ammonia containing species.[6]

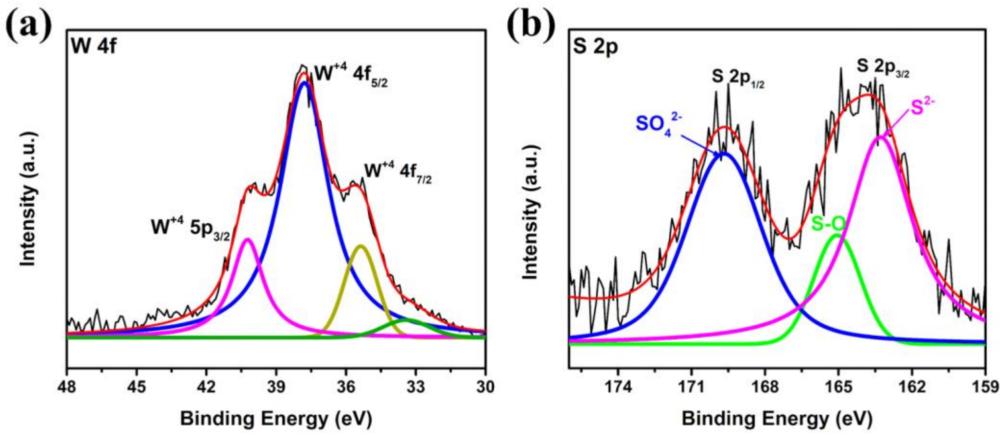

Figure S6. High-resolution XPS spectra of PBA(cage)-WS_2 (a) W 4f, and (b) S 2p.

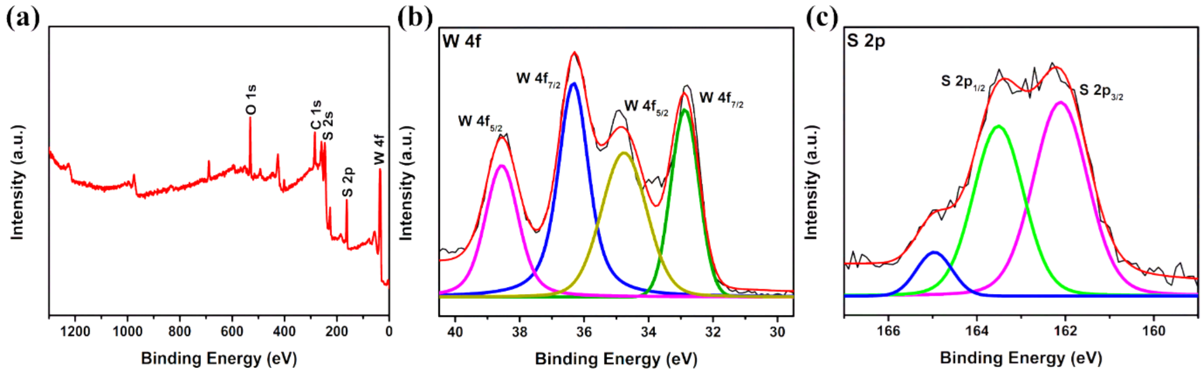

Figure S7. (a) XPS survey spectrum of WS_2; high-resolution XPS spectra of (b) W 4f; and (c) S 2p. Note: The observed 1s carbon peak stems from contamination from the XPS instrument.

The PBA's BE peaks at 781.4 and 797.8 eV in **Figure 4a** are ascribed to the Co^{3+} $2p_{3/2}$ and $2p_{1/2}$, respectively.[7] The BE peaks at 782.8 and 798.9 eV belong to Co^{2+} $2p_{3/2}$ and $2p_{1/2}$, respectively.[7,8] The satellite peaks at around 789.9 and 785.6 eV are two shakeup-type peaks of Co at the high binding energy side of the Co $2p_{3/2}$ edge.[9] Upon etching, the Co^{3+} peaks of PBA(cage) and PBA(cage)-WS_2 disappeared, while the Co^{2+} peaks (Co $2p_{3/2}$ at 782.8 and Co $2p_{1/2}$ at 798.8 eV) increased and broadened. In addition, new satellite peaks appeared for PBA(cage) and PBA(cage)-WS_2 at 804.5 and 803.0, respectively. Evidently, Co^{3+} is reduced to Co^{2+} during the etching and WS_2 growth processes, and the peaks of $[Co^{III}(CN)_6]^{3-}$ at 781.4 and 797.8 eV have disappeared.[10] In **Figure 4b**, the $2p_{3/2}$ Fe peak at 708.8 eV corresponds to Fe^{2+} in PBA as $[Fe(CN)_6]^{4-}$,[11] and it upshifts to 709.6 and 709.9 eV for PBA(cage) and PBA(cage)-WS_2. This shift stems from iron oxidation to Fe^{3+} in $[Fe(CN)_6]^{3-}$, as also evident from the FTIR results.[12] The Fe $2p_{1/2}$ peak at 721.6 eV belongs to Fe^{2+} in PBA,[13] and it respectively shifts to 722.3 and 722.7 eV for PBA(cage) and PBA(cage)-WS_2 due to iron oxide formation.[14] The peaks at 712.3 and 723.7 eV belong to iron oxides and increase upon etching.

Note that the peak at 723.7 eV disappears with the attachment of WS_2, while the peak at 712.5 shifts to 714.4 eV. We attribute this shift to iron sulfidation during the WS_2 formation.[15]

The high-resolution XPS spectra of tungsten and sulfur in PBA(cage)-WS_2 are presented in **Figure S6**. The W $5p_{3/2}$, W $4f_{5/2}$, and W $4f_{7/2}$ XPS peaks are respectively located at 37.7, 35.3, and 33.2 eV, confirming the presence of W^{4+}, **Figure S6a**.[16,17] These peaks are upshifted compared to bare WS_2, whose W $4f_{5/2}$, and W $4f_{7/2}$ peaks are located at 34.8 and 32.7 eV, **Figure S7b**.[18] We attribute this shift to the thin WS_2 layer on the PBA(cage), which is evidently beneficial for OER catalysis. In **Figure S6b**, the S 2p peak of PBA(cage)-WS_2 at 163.1 eV is attributed to the sulfide ion (S^{2-}) of WS_2,[19] and the peak at 169.6 eV is assigned to oxidized S species.[20] The peaks of S $2p_{1/2}$ and $2p_{3/2}$ in the WS_2 nanoflowers, **Figure S7c**, are 163.4 eV and 162.2 eV, respectively.[21]

Table S1. EDX analysis summary of the catalysts' components.

Sample	*Co (at. %)*	*Fe (at. %)*	*W (at. %)*	*S (at. %)*	*Co/Fe*
**PBA(cage)-WS_2**	52.80	39.39	2.48	5.33	1.34
**PBA(cage)**	56.85	43.15	-	-	1.32
**PBA-WS_2**	53.73	37.84	2.46	5.97	1.42
**WS_2**	-	-	28.31	71.69	-
**PBA**	57.23	42.77	-	-	1.46

Table S2. ICP-OES analysis results of the various catalysts.

Sample	*Co (at. %)*	*Fe (at. %)*	*W (at. %)*	*Co/Fe*
**PBA(cage)-WS_2**	54.04	36.98	8.48	1.46
**PBA(cage)**	57.96	42.04	-	1.38
**PBA-WS_2**	55.25	37.25	7.5	1.48
**PBA**	59.33	40.67	-	1.46

PBA oxidation

To verify the source of the catalyst OER activity, the PBA was oxidized before the electrochemical measurement. The PBA oxidation was performed by its calcination at 300 °C with a heating rate of 2 °C min^{-1} for 2 h in air to transform it into metal oxides as adopted from Kuo-Chuan et al.[22] The LSV curve for the PBA oxide in GCE is depicted in **Figure S8**.

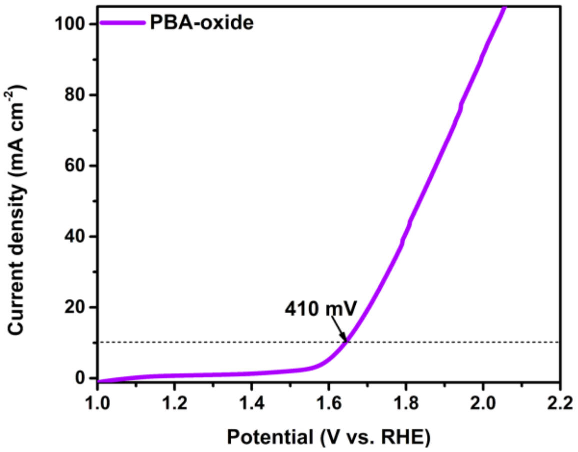

Figure S8. An LSV curve of oxidized PBA in 1.0 M KOH at 10 mVs^{-1}.

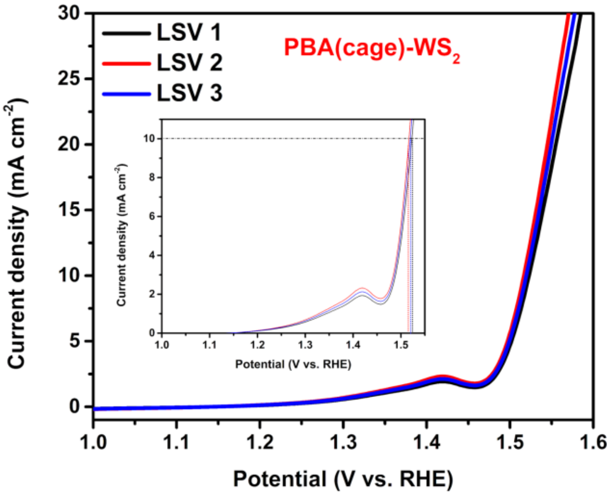

Figure S9. The reproducibility of the LSV curve of PBA(cage)-WS_2.

Table S3. OER performance of PBA(cage)-WS_2 from different experiments

Experiment #	Overpotential
1	293
2	290
3	286
Average ± Standard deviation	290 ± 3

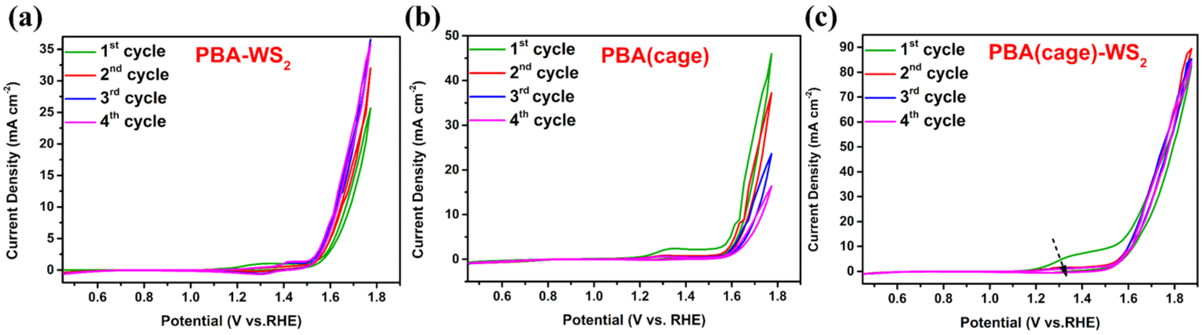

Figure S10. Consecutive CV cycles (10 mV S^{-1}) of (a) PBA-WS_2, (b)PBA(cage), and (c) PBA(cage)-WS_2 in 1.0 M KOH.

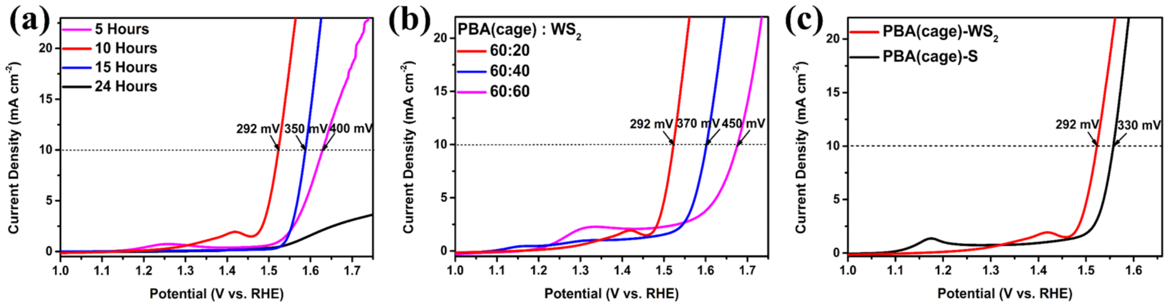

Figure S11. (a) LSV curves of PBA(cage)-WS_2 recorded at different synthesis duration; (b) LSV curves of PBA(cage)-WS_2 with a different mass ratio of PBA(cage):WS_2; (c) LSV curves of PBA(cage)-WS_2 and PBA(cage)-S.

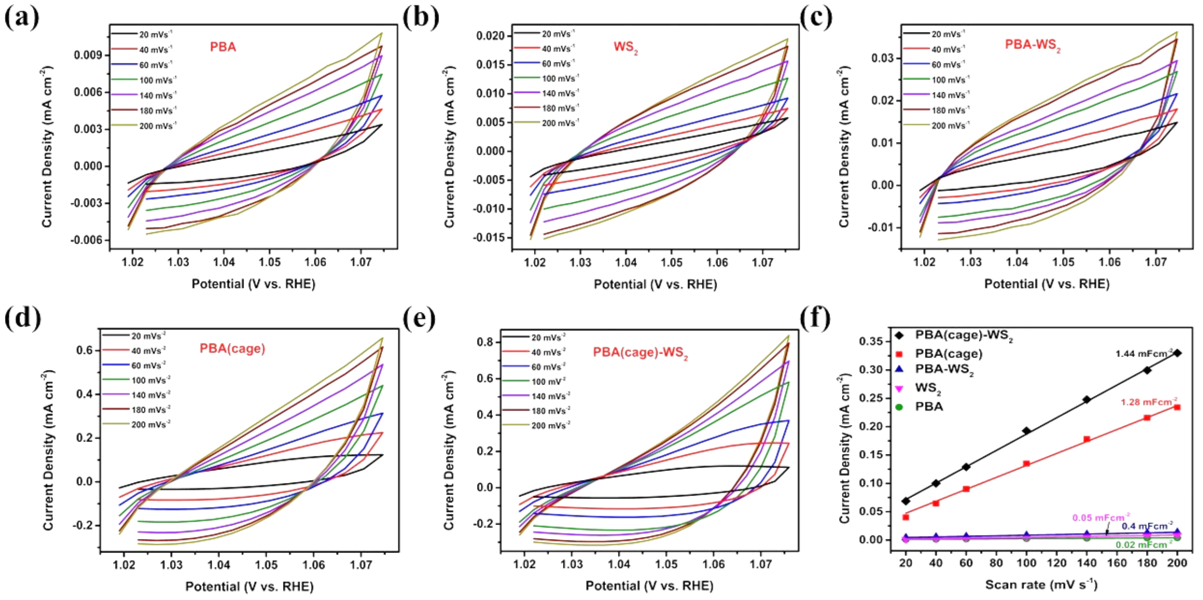

Figure S12. (a-e) Cyclic voltammograms of all the catalysts recorded at different scan rates from 20 to 200 mVs^{-1} in 1.0 M KOH; (f) The C_{dl} derived from $|J_a-J_c|$ vs. scan rate to evaluate electrochemically active surface areas (ECSA).

Table S4. The fitting results of the impedance electrochemical element parameters of various catalysts.

Electrocatalysts	R_s	R_{ct}	Q_{dl}	R_f	Q_f
**PBA(cage)-WS$_2$**	12.13	51.94	0.8444	10.726	0.6800
**PBA(cage)**	12.35	209.80	0.8577	12.710	0.6220
**PBA-WS$_2$**	12.47	458.70	0.9457	4.252	0.8706
**WS$_2$**	15.87	1952.00	0.8174	3.618	0.8989
**PBA**	15.16	5391.12	0.9913	6.983	0.8777

R_s is the uncompensated solution resistance in the equivalent circuit.
Q_{dl} is the constant phase element (CPE) related to the electrochemical double-layer capacitance at the catalyst│electrolyte interface.
R_{ct} is the charge transfer resistance between the interfaces.
Q_f is the CPE associated with the capacitance of the catalyst's thin film.
R_f is the total resistance at the electrode│catalyst interface and charge transfer within the catalyst film.

Table S5: Summarized double-layer capacitance (C_{dl}) and ECSA values of the various electrocatalysts.

Electrocatalysts	C_{dl} (mF/cm^2)	ECSA (cm^2)
**PBA(cage)-W$_2$**	1.44	36.0
**PBA(cage)**	1.28	32.0
**PBA-WS$_2$**	0.4	10.0
**WS$_2$**	0.05	1.25
**PBA**	0.02	0.5

Note: ECSA values were calculated using the equation: ECSA = C_{dl} / C_s, where C_s is the specific capacitance. We used a general specific capacitance value of 40 μF cm^{-2} (in 1.0 M KOH) based on the previous reports[23–25] to calculate ECSA values of all catalysts deposited on glassy carbon electrodes.

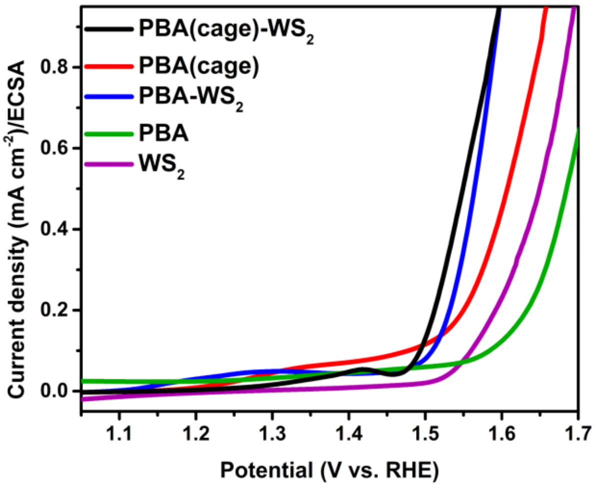

Figure S13. A plot of LSV curves normalized with the electrochemical active surface area (ECSA)

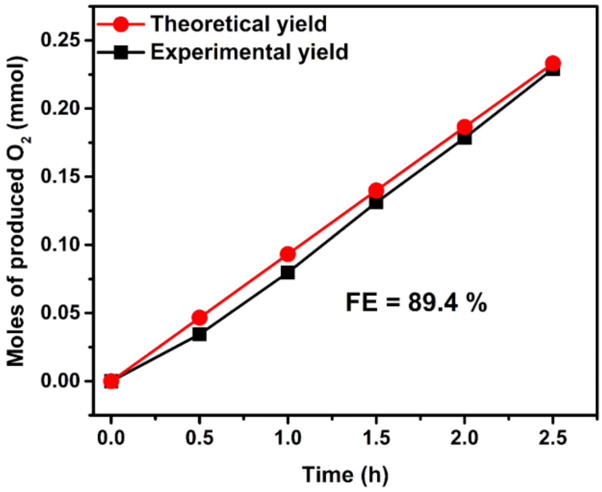

Figure S14. Faradaic efficiency of PBA(cage)-WS_2 for the theoretically calculated and experimentally measured O_2 at a current density of 10 mA cm^{-2}.

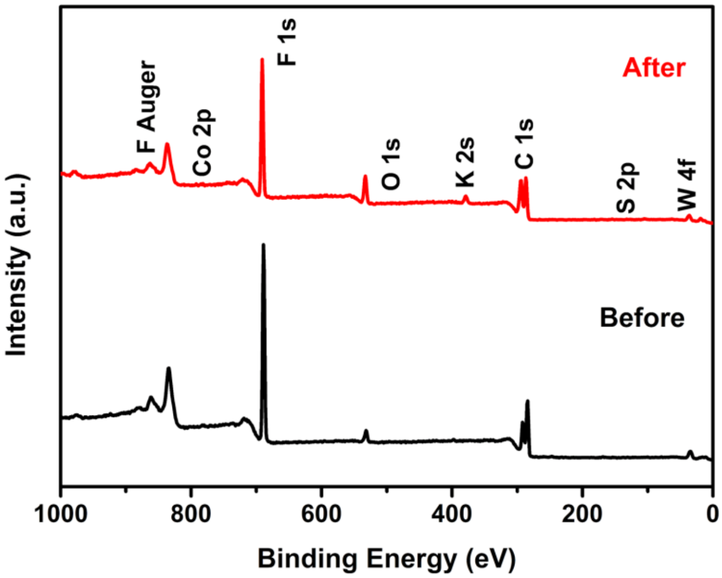

Figure S15. XPS survey spectra of PBA(cage)-WS_2 before and after stability measurement.

Table S6. EDX analysis of Co, Fe, W, and O as determined after 1,000 cycles.

PBA(cage)-WS_2	Before Stability	After Stability
Elements	***Atomic %***	***Atomic %***
**Co**	41.8	38.1
**Fe**	35.3	30.4
**W**	2.3	0.8
**O**	20.6	30.7

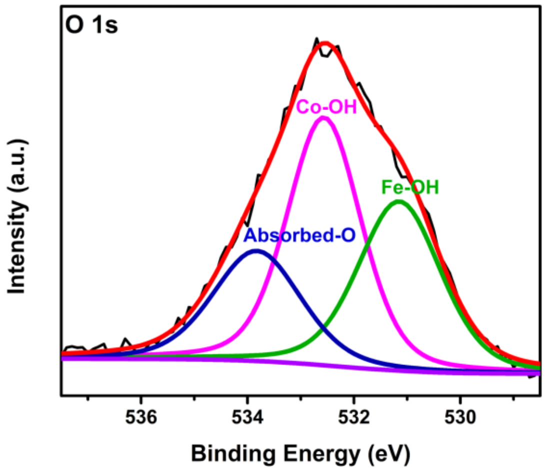

Figure S16. High-resolution XPS spectra of O 1s for PBA(cage)-WS_2 after stability measurement.

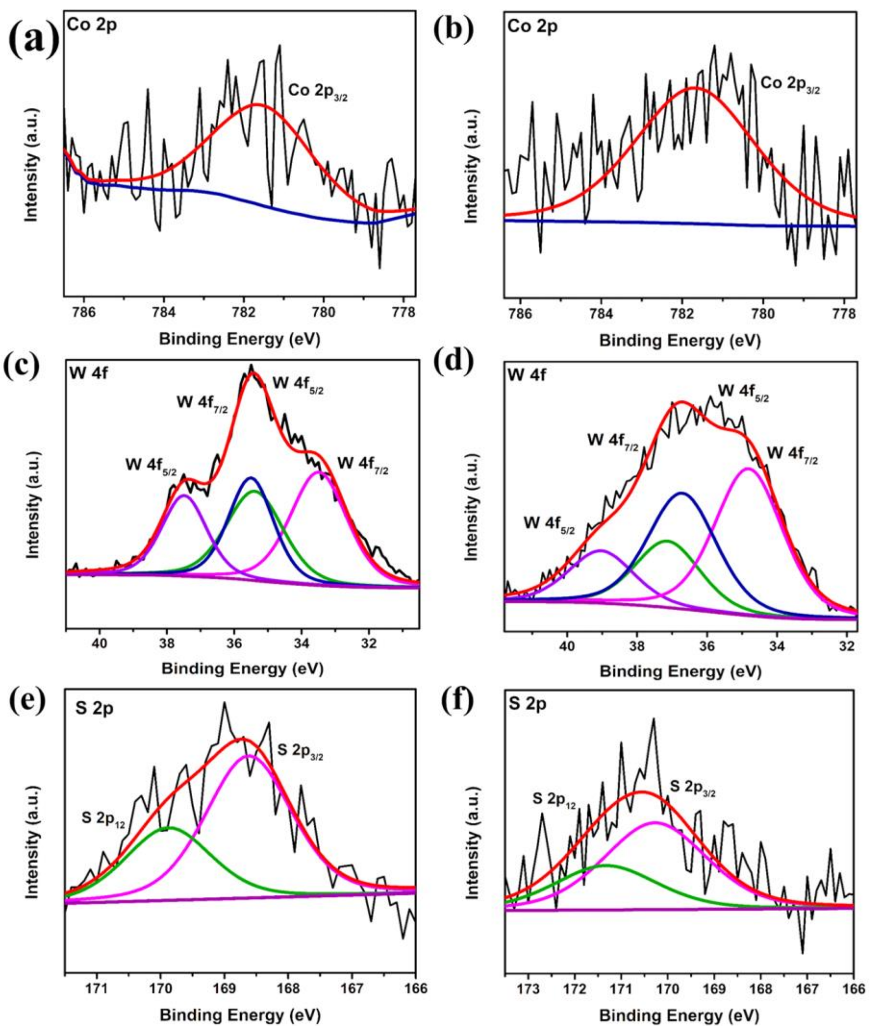

Figure S17. High-resolution XPS spectra of (a) Co 2p (before); (b) Co 2p (after); (c) W 4f (before); (d) W 4f (after), (e) S 2p (before) and (f) S 2p (after) 1,000 cycles for PBA(cage)-WS_2. Note: The catalyst is loaded on carbon cloth, and the ink is prepared using a Nafion binder for which Fe detection through XPS was not possible.

Table S7. Quantitative XPS analysis summary of PBA(cage)-WS_2 before and after cyclic stability.

Elements	Before Stability	After Stability	Before Stability	After Stability
Name	*BE peak (eV)*	*BE peak (eV)*	*Atomic %*	*Atomic %*
**W4f**	35.38	36.68	12.38	5.10
**S2p**	168.5	170.4	11.36	2.88
**O1s**	531.9	532.5	74.30	90.55
**Co2p**	781.5	781.7	1.95	1.48

Table S8. Comparison of OER catalytic performance of the studied catalyst with different reported pristine transition-metal oxides and PBA-oxides

Catalyst	Electrolyte	Substrate	Overpotential	Reference
**Fe-Co oxides *(PBA-oxide)***	1.0 M KOH	CP	380	**26**
**FeCo oxide *(PBA-oxide)***	1.0 M KOH	NF	310	**22**
**Ni_2Fe-O *(PBA-oxide)***	1.0 M KOH	RDE	370	**27**
**$Ni_xCo_{3-x}O_4$ *(PBA-oxide)***	1.0 M KOH	NF	287	**28**
**Co_3O_4 *(PBA-oxide)***	1.0 M KOH	GCE	370	**29**
**CoO**	1.0 M KOH	CFC	346	**30**
**CoO**	1.0 M KOH	FTO	536	**31**
**NiO**	1.0 M KOH	FTO	520	**31**
**NiCoO**	1.0 M KOH	FTO	460	**31**
**Fe-incorporated CoO**	1.0 M KOH		304	**32**
**Mn-Co_3O_4**	1.0 M KOH	CFC	330	**33**
**Se/Ni-Co_3O_4**	1.0 M KOH	CP	290	**34**
**$Fe_{0.1}Ni_{0.9}O$**	0.5 M KOH	Au electrode	297	**35**
**Co_3O_4**	1.0 M KOH	GCE	380	**36**
**Co_3O_4 nanosheets**	0.1 M KOH	Ti foil	300	**37**
**$NiCo_2O_4$**	1.0 M KOH	FTO	323	**38**
**Co_2FeO_4**	1.0 M KOH	RDE	359	**39**
**$Co_{0.5}Fe_{0.5}O$**	1.0 M KOH	CFC	316	**30**

**$Co_{0.37}Ni_{0.26}Fe_{0.37}O$**	1.0 M KOH	CFC	299	**30**
**$CoFe_2O_4$**	1.0 M KOH	RDE	432	**39**
**$NiFe_2O_4$**	1.0 M KOH	CP	381	**40**
**$CoFe_2O_4$**	1.0 M KOH	CP	473	**40**
**$Fe_{0.5}Ni_{0.5}Co_2O_4$**	1.0 M KOH	NF	350	**41**
**PBA(cage)-WS_2**	1.0 M KOH	GCE	290	**This Work**

* Note that catalysts on high surface are supports show improved performances compared to catalysts on a glassy carbon electrode (GCE). However, the improve in the activity is not a catalyst intrinsic property.

References

(1) Ibn Shamsah, S. M. Earth-Abundant Electrocatalysts for Water Splitting: Current and Future Directions. *Catalysts* **2021**, *11* (4), 429. https://doi.org/10.3390/catal11040429.

(2) Roy, A.; Kang, K.-M.; Nah, Y.-C.; La, M.; Choi, D.; Park, S. J. Improved Electrocatalytic Water Oxidation with Cobalt Hydroxide Nano-Flakes Supported on Copper-Modified Nickel Foam. *Electrochim. Acta* **2021**, *383*, 138368. https://doi.org/10.1016/j.electacta.2021.138368.

(3) Martínez-García, R.; Knobel, M.; Goya, G.; Gimenez, M. C.; Romero, F. M.; Reguera, E. Heat-Induced Charge Transfer in Cobalt Iron Cyanide. *J. Phys. Chem. Solids* **2006**, *67* (11), 2289–2299. https://doi.org/10.1016/j.jpcs.2006.05.045.

(4) Cichosz, S.; Masek, A. IR Study on Cellulose with the Varied Moisture Contents: Insight into the Supramolecular Structure. *Materials (Basel).* **2020**, *13* (20), 4573. https://doi.org/10.3390/ma13204573.

(5) Nai, J.; Lou, X. W. (David). Hollow Structures Based on Prussian Blue and Its Analogs for Electrochemical Energy Storage and Conversion. *Adv. Mater.* **2019**, *31* (38), 1706825. https://doi.org/10.1002/adma.201706825.

(6) Nai, J.; Lu, Y.; Yu, L.; Wang, X.; Lou, X. W. D. Formation of Ni-Fe Mixed Diselenide Nanocages as a Superior Oxygen Evolution Electrocatalyst. *Adv. Mater.* **2017**, *29* (41), 1703870. https://doi.org/10.1002/adma.201703870.

(7) Feng, Y.; Wang, X.; Dong, P.; Li, J.; Feng, L.; Huang, J.; Cao, L.; Feng, L.; Kajiyoshi, K.; Wang, C. Boosting the Activity of Prussian-Blue Analogue as Efficient Electrocatalyst for Water and Urea Oxidation. *Sci. Rep.* **2019**, *9* (1), 1–11. https://doi.org/10.1038/s41598-019-52412-1.

(8) Wang, X.; Tian, S.; Zhang, X.; Li, G.; Liu, Y.; Chen, B.; Cheng, B. 3D Ni_3S_2@Mn-Co-OH Cross-Linked Nanosheets on Ni Foam for High Performance Supercapacitor. *Ionics (Kiel).* **2019**, *25* (11), 5485–5494. https://doi.org/10.1007/s11581-019-03089-z.

(9) Nai, J.; Zhang, J.; Lou, X. W. (David). Construction of Single-Crystalline Prussian

Blue Analog Hollow Nanostructures with Tailorable Topologies. *Chem* **2018**, *4* (8), 1967–1982. https://doi.org/10.1016/j.chempr.2018.07.001.

(10) Oku, M.; Hirokawa, K. X-Ray Photoelectron Spectroscopy of Co_3O_4, Fe_3O_4, Mn_3O_4, and Related Compounds. *J. Electron Spectros. Relat. Phenomena* **1976**, *8* (5), 475–481. https://doi.org/10.1016/0368-2048(76)80034-5.

(11) Yatsimirskii, K. B.; Nemoshkalenko, V. V.; Nazarenko, Y. P.; Aleshin, V. G.; Zhilinskaya, V. V.; Tomashevsky, N. A. Use of X-Ray Photoelectron and Mössbauer Spectroscopies in the Study of Iron Pentacyanide Complexes. *J. Electron Spectros. Relat. Phenomena* **1977**, *10* (3), 239–245. https://doi.org/10.1016/0368-2048(77)85023-8.

(12) Vannerberg, N. G. Esca-Spectra of Sodium and Potassium Cyanide and of Sodium and Potassium-Salts of Hexacyanometallates of 1st Transition-Metal Series. *Chem. Scr.* **1976**, *9* (3), 122.

(13) Ma, Y.; Ma, Y.; Dreyer, S. L.; Wang, Q.; Wang, K.; Goonetilleke, D.; Omar, A.; Mikhailova, D.; Hahn, H.; Breitung, B.; Brezesinski, T. High-Entropy Metal–Organic Frameworks for Highly Reversible Sodium Storage. *Adv. Mater.* **2021**, *33* (34), 2101342. https://doi.org/10.1002/adma.202101342.

(14) Rambu, A. P.; Doroftei, C.; Ursu, L.; Iacomi, F. Structure and Gas Sensing Properties of Nanocrystalline Fe-Doped ZnO Films Prepared by Spin Coating Method. *J. Mater. Sci.* **2013**, *48* (12), 4305–4312. https://doi.org/10.1007/s10853-013-7245-5.

(15) Siriwardane, R. V; Cook, J. M. Interactions of SO_2 with Sodium Deposited on Silica. *J. Colloid Interface Sci.* **1985**, *108* (2), 414–422. https://doi.org/10.1016/0021-9797(85)90280-2.

(16) Chen, Y.-W.; Shie, M.-Y.; Hsiao, C.-H.; Liang, Y.-C.; Wang, B.; Chen, I.-W. P. Synthesis of High-Quality Monolayer Tungsten Disulfide with Chlorophylls and Its Application for Enhancing Bone Regeneration. *npj 2D Mater. Appl.* **2020**, *4* (1), 34. https://doi.org/10.1038/s41699-020-00168-y.

(17) Yuwen, L.; Yu, H.; Yang, X.; Zhou, J.; Zhang, Q.; Zhang, Y.; Luo, Z.; Su, S.; Wang, L. Rapid Preparation of Single-Layer Transition Metal Dichalcogenide Nanosheets via Ultrasonication Enhanced Lithium Intercalation. *Chem. Commun.* **2016**, *52* (3), 529–532. https://doi.org/10.1039/C5CC07301D.

(18) Xie, X.; McCarley, R. E. Synthesis, Structure, and Characterization of N-Ligated Tungsten Selenide Cluster Complexes $W_6Se_8L_6$. *Inorg. Chem.* **1995**, *34* (24), 6124–6129. https://doi.org/10.1021/ic00128a025.

(19) Bhandavat, R.; David, L.; Singh, G. Synthesis of Surface-Functionalized WS_2 Nanosheets and Performance as Li-Ion Battery Anodes. *J. Phys. Chem. Lett.* **2012**, *3* (11), 1523–1530. https://doi.org/10.1021/jz300480w.

(20) Liu, W.; Benson, J.; Dawson, C.; Strudwick, A.; Raju, A. P. A.; Han, Y.; Li, M.; Papakonstantinou, P. The Effects of Exfoliation, Organic Solvents and Anodic Activation on the Catalytic Hydrogen Evolution Reaction of Tungsten Disulfide. *Nanoscale* **2017**, *9* (36), 13515–13526. https://doi.org/10.1039/C7NR04790H.

(21) Wu, A.; Tian, C.; Yan, H.; Jiao, Y.; Yan, Q.; Yang, G.; Fu, H. Hierarchical MoS_2 @MoP Core–Shell Heterojunction Electrocatalysts for Efficient Hydrogen Evolution Reaction over a Broad PH Range. *Nanoscale* **2016**, *8* (21), 11052–11059. https://doi.org/10.1039/C6NR02803A.

(22) Chuang, C.-H.; Hsiao, L.-Y.; Yeh, M.-H.; Wang, Y.-C.; Chang, S.-C.; Tsai, L.-D.; Ho, K.-C. Prussian Blue Analogue-Derived Metal Oxides as Electrocatalysts for Oxygen Evolution Reaction: Tailoring the Molar Ratio of Cobalt to Iron. *ACS Appl. Energy Mater.* **2020**, *3* (12), 11752–11762. https://doi.org/10.1021/acsaem.0c01903.

(23) Sadaqat, M.; Manzoor, S.; Nisar, L.; Hassan, A.; Tyagi, D.; Shah, J. H.; Ashiq, M. N.; Joya, K. S.; Alshahrani, T.; Najam-ul-Haq, M. Iron Doped Nickel Ditelluride Hierarchical Nanoflakes Arrays Directly Grown on Nickel Foam as Robust Electrodes for Oxygen Evolution Reaction. *Electrochim. Acta* **2021**, *371*, 137830. https://doi.org/10.1016/j.electacta.2021.137830.

(24) Browne, M. P.; Nolan, H.; Duesberg, G. S.; Colavita, P. E.; Lyons, M. E. G. Low-Overpotential High-Activity Mixed Manganese and Ruthenium Oxide Electrocatalysts for Oxygen Evolution Reaction in Alkaline Media. *ACS Catal.* **2016**, *6* (4), 2408–2415. https://doi.org/10.1021/acscatal.5b02069.

(25) McCrory, C. C. L.; Jung, S.; Peters, J. C.; Jaramillo, T. F. Benchmarking Heterogeneous Electrocatalysts for the Oxygen Evolution Reaction. *J. Am. Chem. Soc.* **2013**, *135* (45), 16977–16987. https://doi.org/10.1021/ja407115p.

(26) Ishizaki, M.; Fujii, H.; Toshima, K.; Tanno, H.; Sutoh, H.; Kurihara, M. Preparation of Co-Fe Oxides Immobilized on Carbon Paper Using Water-Dispersible Prussian-Blue Analog Nanoparticles and Their Oxygen Evolution Reaction (OER) Catalytic Activities. *Inorganica Chim. Acta* **2020**, *502*, 119345. https://doi.org/10.1016/j.ica.2019.119345.

(27) Xie, Z.; Zhang, C.; He, X.; Liang, Y.; Meng, D.; Wang, J.; Liang, P.; Zhang, Z. Iron and Nickel Mixed Oxides Derived From $Ni^{II}Fe^{II}$-PBA for Oxygen Evolution Electrocatalysis. *Front. Chem.* **2019**, *7*. https://doi.org/10.3389/fchem.2019.00539.

(28) Shen, Y.; Guo, S.-G.; Du, F.; Yuan, X.-B.; Zhang, Y.; Hu, J.; Shen, Q.; Luo, W.; Alsaedi, A.; Hayat, T.; Wen, G.; Li, G.-L.; Zhou, Y.; Zou, Z. Prussian Blue Analogue-Derived Ni and Co Bimetallic Oxide Nanoplate Arrays Block-Built from Porous and Hollow Nanocubes for the Efficient Oxygen Evolution Reaction. *Nanoscale* **2019**, *11* (24), 11765–11773. https://doi.org/10.1039/C9NR01804B.

(29) Feng, Y.; Yu, X.-Y.; Paik, U. Formation of Co_3O_4 Microframes from MOFs with Enhanced Electrochemical Performance for Lithium Storage and Water Oxidation. *Chem. Commun.* **2016**, *52* (37), 6269–6272. https://doi.org/10.1039/C6CC02093C.

(30) Chen, W.; Wang, H.; Li, Y.; Liu, Y.; Sun, J.; Lee, S.; Lee, J.-S.; Cui, Y. In Situ Electrochemical Oxidation Tuning of Transition Metal Disulfides to Oxides for Enhanced Water Oxidation. *ACS Cent. Sci.* **2015**, *1* (5), 244–251. https://doi.org/10.1021/acscentsci.5b00227.

(31) Ali, A.; Najaf, D.; Nazir, A.; Haider, A.; Iqbal, M.; Alwadai, N.; Kausar, A.; Ahmad, A. Fabrication of Efficient Electrocatalysts for Electrochemical Water Oxidation Using Bimetallic Oxides System. *ACS Omega* **2023**, *8* (10), 9539–9546. https://doi.org/10.1021/acsomega.2c08288.

(32) Lyu, F.; Bai, Y.; Wang, Q.; Wang, L.; Zhang, X.; Yin, Y. Coordination-Assisted Synthesis of Iron-Incorporated Cobalt Oxide Nanoplates for Enhanced Oxygen Evolution. *Mater. Today Chem.* **2019**, *11*, 112–118. https://doi.org/10.1016/j.mtchem.2018.10.010.

(33) Qi, J.; Wang, H.; Lin, J.; Li, C.; Si, X.; Cao, J.; Zhong, Z.; Feng, J. Mn and S Dual-

Doping of MOF-Derived Co_3O_4 Electrode Array Increases the Efficiency of Electrocatalytic Generation of Oxygen. *J. Colloid Interface Sci.* **2019**, *557*, 28–33. https://doi.org/10.1016/j.jcis.2019.09.009.

(34) Li, R.; Guo, Y.; Chen, H.; Wang, K.; Tan, R.; Long, B.; Tong, Y.; Tsiakaras, P.; Song, S.; Wang, Y. Anion–Cation Double Doped Co_3O_4 Microtube Architecture to Promote High-Valence Co Species Formation for Enhanced Oxygen Evolution Reaction. *ACS Sustain. Chem. Eng.* **2019**, *7* (13), 11901–11910. https://doi.org/10.1021/acssuschemeng.9b02558.

(35) Fominykh, K.; Chernev, P.; Zaharieva, I.; Sicklinger, J.; Stefanic, G.; Döblinger, M.; Müller, A.; Pokharel, A.; Böcklein, S.; Scheu, C.; Bein, T.; Fattakhova-Rohlfing, D. Iron-Doped Nickel Oxide Nanocrystals as Highly Efficient Electrocatalysts for Alkaline Water Splitting. *ACS Nano* **2015**, *9* (5), 5180–5188. https://doi.org/10.1021/acsnano.5b00520.

(36) Chen, S.; Zhao, Y.; Sun, B.; Ao, Z.; Xie, X.; Wei, Y.; Wang, G. Microwave-Assisted Synthesis of Mesoporous Co_3O_4 Nanoflakes for Applications in Lithium Ion Batteries and Oxygen Evolution Reactions. *ACS Appl. Mater. Interfaces* **2015**, *7* (5), 3306–3313. https://doi.org/10.1021/am508136k.

(37) Xu, L.; Jiang, Q.; Xiao, Z.; Li, X.; Huo, J.; Wang, S.; Dai, L. Plasma-Engraved Co_3O_4 Nanosheets with Oxygen Vacancies and High Surface Area for the Oxygen Evolution Reaction. *Angew. Chemie Int. Ed.* **2016**, *55* (17), 5277–5281. https://doi.org/10.1002/anie.201600687.

(38) Shi, H.; Zhao, G. Water Oxidation on Spinel $NiCo_2O_4$ Nanoneedles Anode: Microstructures, Specific Surface Character, and the Enhanced Electrocatalytic Performance. *J. Phys. Chem. C* **2014**, *118* (45), 25939–25946. https://doi.org/10.1021/jp508977j.

(39) Xiang, W.; Yang, N.; Li, X.; Linnemann, J.; Hagemann, U.; Ruediger, O.; Heidelmann, M.; Falk, T.; Aramini, M.; DeBeer, S.; Muhler, M.; Tschulik, K.; Li, T. 3D Atomic-Scale Imaging of Mixed Co-Fe Spinel Oxide Nanoparticles during Oxygen Evolution Reaction. *Nat. Commun.* **2022**, *13* (1), 179. https://doi.org/10.1038/s41467-021-27788-2.

(40) Maruthapandian, V.; Mathankumar, M.; Saraswathy, V.; Subramanian, B.; Muralidharan, S. Study of the Oxygen Evolution Reaction Catalytic Behavior of $Co_xNi_{1-x}Fe_2O_4$ in Alkaline Medium. *ACS Appl. Mater. Interfaces* **2017**, *9* (15), 13132–13141. https://doi.org/10.1021/acsami.6b16685.

(41) Yan, K.-L.; Shang, X.; Li, Z.; Dong, B.; Li, X.; Gao, W.-K.; Chi, J.-Q.; Chai, Y.-M.; Liu, C.-G. Ternary Mixed Metal Fe-Doped $NiCo_2O_4$ Nanowires as Efficient Electrocatalysts for Oxygen Evolution Reaction. *Appl. Surf. Sci.* **2017**, *416*, 371–378. https://doi.org/10.1016/j.apsusc.2017.04.204.
